# CTHRC1 induces non-small cell lung cancer (NSCLC) invasion through upregulating MMP-7/MMP-9

**DOI:** 10.1186/s12885-018-4317-6

**Published:** 2018-04-10

**Authors:** Weiling He, Hui Zhang, Yuefeng Wang, Yanbin Zhou, Yifeng Luo, Yongmei Cui, Neng Jiang, Wenting Jiang, Han Wang, Di Xu, Shuhua Li, Zhuo Wang, Yangshan Chen, Yu Sun, Yang Zhang, Hsian-Rong Tseng, Xuenong Zou, Liantang Wang, Zunfu Ke

**Affiliations:** 1 Department of Pathology, The First Affiliated Hospital, Sun Yat-sen University, No. 58, ZhongShan Second Road, Guangdong, 510080 China; 2 Department of Gastrointestinal Surgery, The First Affiliated Hospital, Sun Yat-sen University, No. 58, ZhongShan Second Road, Guangdong, 510080 China; 3 Department of Respiratory Medicine, The First Affiliated Hospital, Sun Yat-sen University, No. 58, ZhongShan Second Road, Guangdong, 510080 China; 4 Department of Thoracic Surgery, The Central Hospital of Wuhan, No.26 Shenli Street, Jiang’an District, Wuhan, 430014 Hubei Province China; 5 Biomedical Engineering, The University of Texas at El Paso, El Paso, TX USA; 6 Department of Molecular and Medical Pharmacology, Crump Institute for Molecular Imaging (CIMI), California NanoSystems Institute (CNSI), University of California, Los Angeles, 570 Westwood Plaza, California, Los Angeles 90095-1770 USA; 7 Guangdong Provincial Key Laboratory of Orthopedics and Traumatology, The First Affiliated Hospital, Sun Yat-sen University, No. 58, ZhongShan Second Road, Guangdong, 510080 China

**Keywords:** Lung cancer, CTHRC1, MMP7, MMP9, Invasion/metastasis

## Abstract

**Background:**

The strong invasive and metastatic nature of non-small cell lung cancer (NSCLC) leads to poor prognosis. Collagen triple helix repeat containing 1 (CTHRC1) is involved in cell migration, motility and invasion. The object of this study is to investigate the involvement of CTHRC1 in NSCLC invasion and metastasis.

**Methods:**

A proteomic analysis was performed to identify the different expression proteins between NSCLC and normal tissues. Cell lines stably express CTHRC1, MMP7, MMP9 were established. Invasion and migration were determined by scratch and transwell assays respectively. Clinical correlations of CTHRC1 in a cohort of 230 NSCLC patients were analysed.

**Results:**

CTHRC1 is overexpressed in NSCLC as measured by proteomic analysis. Additionally, CTHRC1 increases tumour cell migration and invasion in vitro. Furthermore, CTHRC1 expression is significantly correlated with matrix metalloproteinase (MMP)7 and MMP9 expression in sera and tumour tissues from NSCLC. The invasion ability mediated by CTHRC1 were mainly MMP7- and MMP9-dependent. MMP7 or MMP9 depletion significantly eradicated the pro-invasive effects mediated by CTHRC1 on NSCLC cells. Clinically, patients with high CTHRC1 expression had poor survival.

**Conclusions:**

CTHRC1 serves as a pro-metastatic gene that contributes to NSCLC invasion and metastasis, which are mediated by upregulated MMP7 and MMP9 expression. Targeting CTHRC1 may be beneficial for inhibiting NSCLC metastasis.

**Electronic supplementary material:**

The online version of this article (10.1186/s12885-018-4317-6) contains supplementary material, which is available to authorized users.

## Background

Lung cancer is one of the most common malignant tumours and remains the leading cause of cancer-related death in China and around the world [1]. Among all the lung cancers, non-small cell lung cancer (NSCLC) is the most common and aggressive type, accounting for ~ 85% of cases [2, 3]. Surgical resection remains the preferred clinical treatment for NSCLC patients in the early stages of disease. Despite advances in radio- and chemotherapy and the development of new targeted therapies in the past few years, 5-year survival rate remains poor in NSCLC patients due to unresectable advanced or metastatic disease at diagnosis. The high mortality and low cure rates for NSCLC are largely attributed to the strong ability of lung cancer cells to invade surrounding tissue or metastasize to other remote sites [4–6]. Hence, understanding the molecular mechanisms underlying NSCLC invasion and metastasis is essential.

Tumor metastasis is a complex process involving cell adhesion and proteolytic degradation of the extracellular matrix (ECM) [7, 8]. Matrix metalloproteinases (MMPs) are characterized by their ability to degrade extracellular matrix (ECM) proteins and expose cryptic sites within the matrix molecules to facilitate tumour invasion and metastasis [9–12]. A previous study has shown MMP7 in promoting ovarian cancer cell invasion [13]. Additionally, mice deficient in MMP9 are resistant to tumour metastasis [14]. MMP9 is highly involved in strengthening the invasion capability of NSCLC [15]. Clinically, MMP7 and MMP9 expression correlates with poor prognosis of NSCLC [16–18]. To further understand MMP modulation mechanisms in NSCLC to search for new therapeutic targets is thus imperative.

Collagen triple helix repeat containing 1 (CTHRC1) was originally identified in balloon-injured rat arteries, and its overexpression in fibroblasts is associated with increased cell migration, motility and invasion [19]. CTHRC1 is widely upregulated in several solid tumours, including melanoma and cancers of the gastrointestinal tract, breast, thyroid, liver and pancreas [20]. Furthermore, recombinant CTHRC1 protein augments the migration and invasion capacities of primary gastrointestinal stromal tumours [21]. According to Chen et al. [22], CTHRC1 promotes tumour invasion and predicts poor prognosis in hepatocellular carcinoma. In our previous study, CTHRC1 overexpression in NSCLC cells was associated with tumour aggressiveness [23]; however, how CTHRC1 is involved in tumour cell migration and metastasis has yet to be fully elucidated. In a recent study by Park et al. [24], CTHRC1 regulated pancreatic cancer migration and adhesion by inducing Src, MEK and Rac1 activation. We previously showed the ability of CTHRC1 to increase the invasive capability of epithelial ovarian cancer cells by provoking constitutive activation of Wnt/β-catenin signalling [25]. However, whether CTHRC1 is involved in cancer cell invasion and metastasis has not been completely clarified. Additionally, the mechanisms employed by CTHRC1 to regulate MMPs remain uncharacterized by previous investigations.

In our present study, we detected CTHRC1 overexpression in NSCLC tissues by performing a proteomic analysis and further confirmed the results through western blotting and in IHC assays at the tissue and cell levels. Furthermore, IHC analysis revealed a close relationship between CTHRC1 overexpression and lymph node metastasis, clinical stage and overall survival. Moreover, we demonstrated that CTHRC1 promoted tumour invasion by regulating MMP7 and MMP9 expression, which is mediated by the AP-1/c-Jun and NF-κB pathways, respectively. Additionally, serum concentration of CTHRC1 correlates with metastasis, clinical stage and circulating tumour cell (CTC) number and functions as an important prognostic factor for NSCLC patients. In summary, our findings represent an important step forward in understanding the role of CTHRC1 in NSCLC metastasis.

## Methods

### Patient and tissue information

Sera and primary tumour tissues were collected from a total of 230 cases of clinically and immunohistologically verified NSCLC (obtained from 2006 to 2011) identified in the pathology archives of the Affiliated First Hospital, Sun Yat-sen University, and the Central Hospital of Wuhan. NSCLC was verified by performing haematoxylin and eosin (HE) staining and immunohistochemistry as shown in Additional file 1: Figure S1A. Patients’ clinical characteristics are listed in Additional file 1: Table S1.

### Proteomic analysis

Proteomic analysis was performed**.** NSCLC tissues (*n* = 20) and adjacent non-tumour tissues (*n* = 20) were used to extract proteins for analysis. Tumour tissues were homogenized in lysis buffer via sonication on ice. A 2-D CleanQ2-Up Kit (Amersham Biosciences, UK) and a 2-D Quant Kit (GE Healthcare, London, UK) were used for protein purification and concentration qualification, respectively, according to the manufacturers’ instructions. Two-dimensional gel electrophoresis was performed using an immobilized pH gradient (IPG) strip (24 cm, pH 3–10 NL; GE Healthcare), in which proteins were separated according to their isoelectric point (pI) and molecular weight. Visualized stained proteins were selected using an Ettan Spot Handling Workstation (GE Healthcare), and spots of interest were digested with trypsin. Peptide mass mapping was performed via matrix-assisted laser desorption time-of-flight mass spectrometry (MALDI-TOF MS) using an ABI Voyager DE-STR mass spectrometer. The MASCOT Database (http://www.matrixscience.com/search_form_select.html) was employed to identify the original proteins. The search criteria were as follows: *Homo sapiens*, trypsin cleavage, and no constraints on either the molecular weight or the isoelectric point of the protein.

### Western blot

After electrophoresis, Membranes were incubated with primary antibodies in 5% milk/TBST at 4 °C overnight. The membranes were then washed with TBST and subsequently incubated with HRP-conjugated anti-rabbit IgG at room temperature for 60 min. Membranes were washed with TBST, and signals were detected via enhanced chemiluminescence (ECL). Cell nucleoprotein was extracted using EpiQuik Nuclear Extraction Kit (Epigentek, Farmingdale, NY) according to the manufacturer’s instructions.

### Reverse dot hybridization

All assays were performed as previously described [26] and in strict accordance with the instructions provided with the kit (Roche, USA). Specific probes targeting MMP genes were designed and listed in Additional file 1: Table S2. siRNA sequences and RT-PCR primers are listed in Additional file 1: Tables S3 and S4 respectively.

### Luciferase reporter gene assay

NCI-H1975 cells were seeded in 24-well plates in triplicate and allowed to adhere for 24 h. Luciferase reporter plasmids (200 ng) containing different fragments of the MMP7 or MM9 promoters were transfected into cells using Lipofectamine 2000 with 1 ng of pRL-SV40 Renilla luciferase as an internal control. Cell extracts were prepared 24 h after transfection, and luciferase signals were measured using the Dual-Luciferase Reporter Assay System (Promega, USA) according to the manufacturer’s instructions.

### Chromatin immunoprecipitation (ChIP)

ChIP was performed using a Chromatin Immunoprecipitation Kit (Upstate) according to the manufacturer’s instructions. Briefly, NCI-H1975 cells were treated with 1% formaldehyde to cross-link proteins to DNA in a 100-mm culture dish. Sonication was applied to the cell debris to shear DNA into 300–1000-bp fragments. Equal amounts of chromatin supernatants, containing an antibody against CTHRC1 (1 μg) or an equal amount of control IgG, were incubated overnight at 4 °C with shaking. PCR was performed after the reverse cross-linking of protein/DNA complexes to release DNA.

### Immunohistochemistry

Paraffin-embedded tissues were sectioned (4 μm) and incubated with anti-CTHRC-1 (Abcam, Cambridge, UK), anti-MMP7 (Abcam, Cambridge, UK) and anti-MMP9 (Abcam, Cambridge, UK) primary antibodies at 4 °C overnight. After washing with PBS, sections were then incubated with an HRP-conjugated goat anti-rabbit secondary antibody for 1 h at room temperature. Peroxidase was visualized with 3,3′-diaminobenzidine, and haematoxylin was used as a counterstain.

### CTC enrichment using the NanoVelcro system

CTC were detected by NanoVelcro system as we previously described [27]. Blood specimens were collected in EDTA tubes. Blood samples were processed within 24 h. NH_4_Cl was added to whole blood at a ratio of 10:1 *v*/v and incubated for 20 min at room temperature to lyse the red blood cells. Samples were centrifuged at 200 g for 5 min, and the supernatants were removed. Cell pellets were re-suspended. Immunocytochemistry was applied to visualize cells captured on the SiNW substrate. The microchannels were loaded with 100 μl of fluorophore-labelled antibody solution (20 μl/1 ml of the initial concentration) and incubated at 4 °C overnight. CTCs were identified based on positive staining for cytokeratin (PE) and negative staining for CD45 (FITC). An experienced pathologist characterized the phenotypes and morphologies of tumour cells.

### Enzyme-linked immunosorbent assay (ELISA)

Sera were collected from NSCLC patients or healthy controls. The concentrations of CTHRC1 in the sera were measured by ELISA as previous described [28].

### Statistical analysis

All above experiments were performed at least three times. Statistical analysis was carried out using SPSS software (version 16.0; SPSS, Chicago, IL, USA). The χ2 test was applied to analyse the relationships between CTHRC1, MMP7, and MMP9 expression and clinicopathologic parameters. An unpaired, two-tailed Student’s t-test was used to determine the between- group significance. Bivariate correlation analysis was calculated as Spearman’s rank correlation coefficient. Survival curves were plotted using the Kaplan-Meier method and compared with the log-rank test. ROC curve analysis was carried out to determine the CTHRC1 cut-off points for metastasis and recurrence status. *P* values < 0.05 were considered significant.

## Results

### CTHRC1 overexpression in NSCLC tissues correlates with clinical metastasis status in NSCLC

**Fig. 1:**
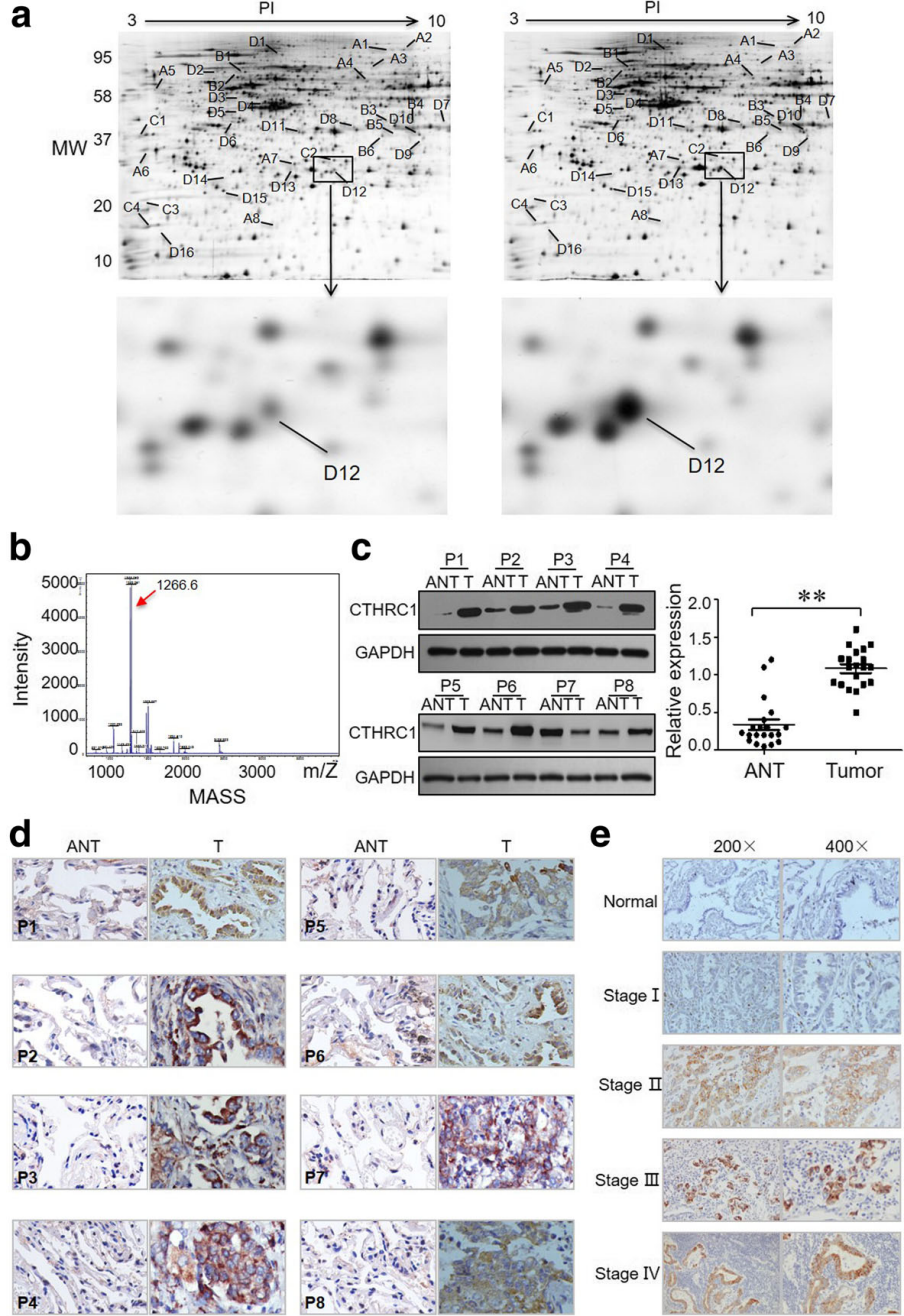
Differential protein expression in NSCLC and corresponding adjacent nontumor tissues (ANTs) samples and the correlation with tumor metastasis. Thirty-four differential protein spots were identified from NSCLC (**a**, right) and ANT (**a**, left) samples in the representative silver-stained 2D gel image, and the outlined areas show CTHRC1 upregulation in NSCLC tissues (*n* = 20). **b** MS identification analysis of CTHRC1. The red arrow marks the specific peak corresponding to the CTHRC1 protein. **c, d** Comparative CTHRC1 protein quantification in paired primary NSCLC tissues (T) and their corresponding ANTs in the left panel (*n* = 20) as measured by western blotting and IHC (**p < 0.01). **e** CTHRC1 levels increase as tumour grade (I–IV) progresses, as determined by IHC staining (*n* = 230). Representative western blot bands and IHC images are presented. Three independent experiments were performed

Comparative proteomic analysis simultaneously revealed 34 differential spots in NSCLC tissues compared with corresponding adjacent non-tumour tissues (ANTs). All protein spots of interest on silver-stain gels (Fig. 1a) were identified by MALDI-TOF/MS and further confirmed via a comparative sequence search in the MASCOT database. The identified proteins are summarized in Additional file 1: Table S5. In general, CTHRC1 was upregulated in all 20 NSCLC individuals. Representative peptide mass fingerprinting (PMF) of CTHRC1 is shown in Fig. 1b. We also confirmed that CTHRC1 is overexpressed in all the NSCLC cell lines (Additional file 1: Figure S1B).

CTHRC1 overexpression is reportedly associated with tumour invasion and metastasis [20, 29]. To investigate whether NSCLC also exhibits strong CTHRC1 expression compared to ANTs, we first measured CTHRC1 expression in twenty paired NSCLC and ANT samples by performing western blotting. Compared to individual corresponding ANTs, CTHRC1 expression was significantly higher in NSCLC tissues (Fig. 1c), which was further confirmed at the RNA level by RT-PCR (Additional file 1: Figure S1C). Consistent with the western blot and RT-PCR results, IHC data further verified the upregulation of CTHRC1 in primary NSCLC tissues (Fig. 1d). Next, we investigated the correlation between CTHRC1 expression and NSCLC metastasis. Pathologically verified NSCLC tumour tissues and ANTs were collected. According to the IHC results, CTHRC1 was expressed at very low levels in normal lung tissue. In contrast, CTHRC1 expression was very high in 55.2% of primary NSCLC tissues, and furthermore, CTHRC1 expression in NSCLC tissues was associated with tumour metastasis (Additional file 1: Table S1). Additionally, compared to early stages, advanced stages characterized by localized invasion or distant metastasis had significantly higher levels of CTHRC1 (Fig. 1e).

### CTHRC1 promotes NSCLC cell migration and invasion

**Fig. 2:**
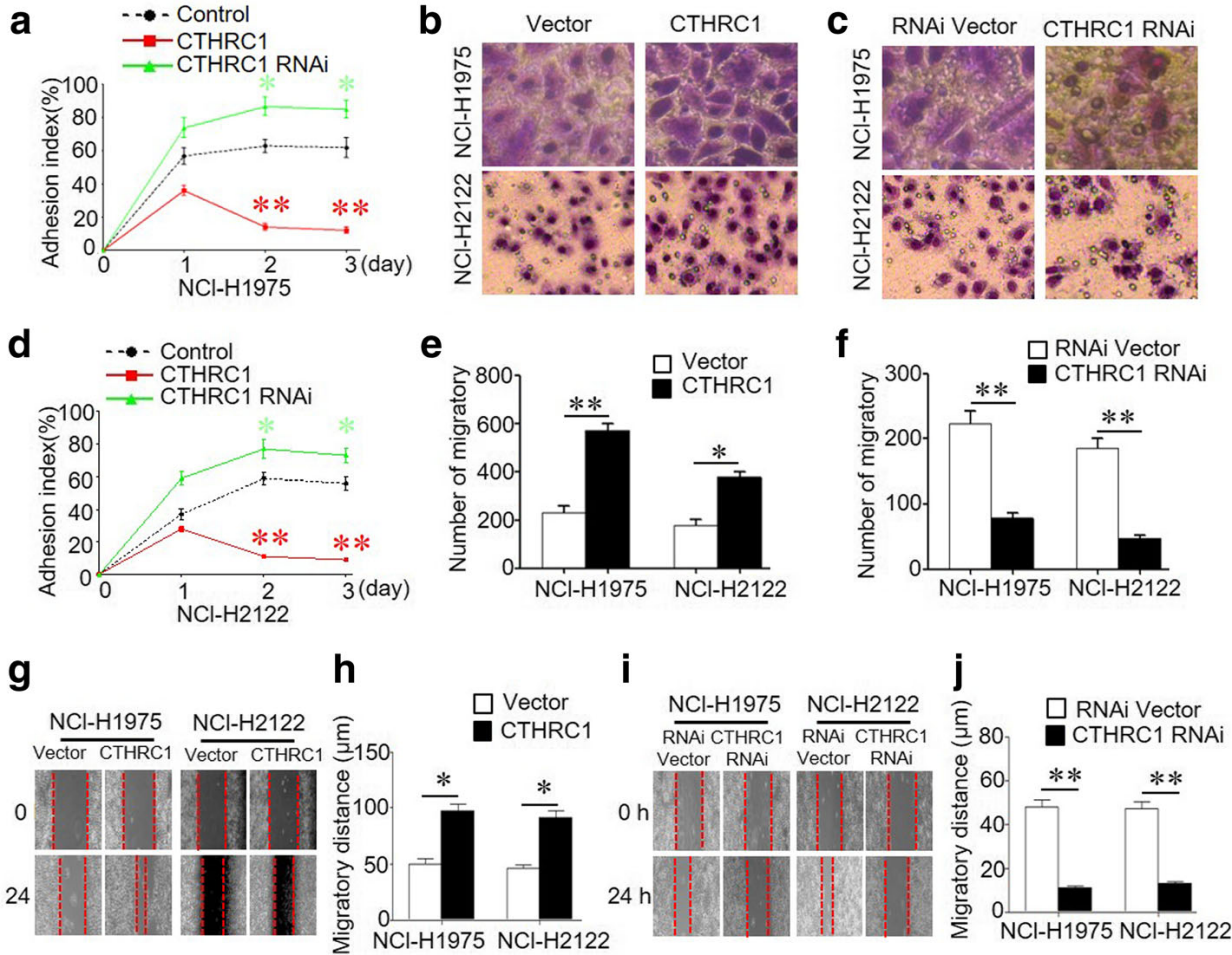
CTHRC1 inhibits adhesion and promotes NSCLC cell migration and invasion of in vitro and metastasis in vivo. **a, d** CTHRC1 overexpression inhibited cell adhesion; CTHRC1 knockdown increased cell adhesion; (**b-f**) Transwell assay results indicated that ectopic overexpression of CTHRC1 promoted cell invasion; knockdown of endogenous CTHRC1 inhibited cell invasion. Migratory cells were stained with HE and qualitatively assessed, as summarized in the bar graphs. **g-j** A wound assay indicated CTHRC1 overexpression increased tumour cell migration; CTHRC1 knockdown inhibited tumour cell migration. Migration distances were measured and are summarized in the bar graphs; *p < 0.05 and **p < 0.01

Based on the above data, CTHRC1 was overexpressed and associated with disease invasion and metastasis in NSCLC. To provide direct evidence supporting the contribution of CTHRC1 to NSCLC invasion and migration, we first selected NCI-H1975 and NCI-H2122 to establish cell lines in which CTHRC1 was stably overexpressed or knocked down. Overexpression or depletion efficiencies were confirmed by performing western blot as shown in Additional file 1: Figure S2. An adhesion assay demonstrated decreased tumour cell adhesion accompanying the ectopic overexpression of CTHRC1, while depletion of CTHRC1 increased tumour cell adhesion (Fig. 2a, d). Additionally, CTHRC1 overexpression increased the ability of tumour cells to invade through a Transwell gel, and CTHRC1 depletion suppressed tumour cell invasion (Fig. 2b, c, e, f). Furthermore, tumour cell migration speed increased with CTHRC1 overexpression but was inhibited with CTHRC1 depletion (Fig. 2g-j).

### CTHRC1 regulates MMP7 and MMP9 expression in vitro and in vivo

**Fig. 3:**
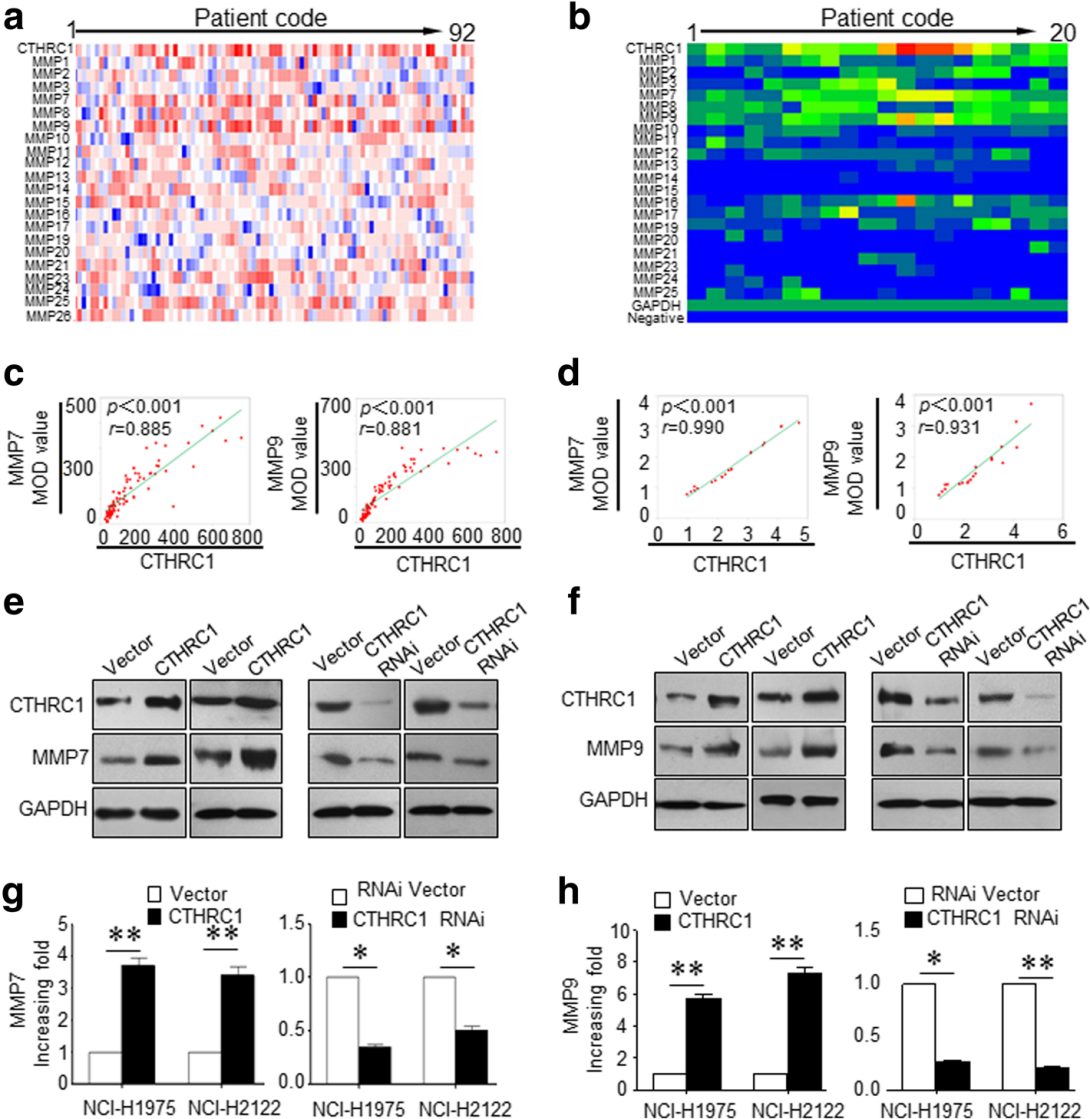
CTHRC1 regulates MMP7 and MMP9 expression in vitro and in vivo. **a** The heat map shows the concentration distributions of CTHRC1 and diverse MMPs in the sera of NSCLC patients as measured by ELISA (*n* = 92). **b** mRNA levels of CTHRC1 and diverse MMPs in NSCLC tissues were further analysed by reverse dot blot hybridization (*n* = 20). **c, d** CTHRC1 expression was positively correlated with MMP7 and MMP9 in both sera and primary tumour tissues. **e, f** Western blot analysis demonstrated increased MMP7 and MMP9 production accompanying the ectopic overexpression of CTHRC1; CTHRC1 knockdown downregulated MMP7 and MMP9 expression. **g, h** The western blot results were semi-quantified and shown in bar graph form. Representative images were shown and all the experiments had been repeated 3 times. *p < 0.05 and **p < 0.01

Based on our data, CTHRC1 regulated the invasion and metastasis of NSCLC in vitro and in vivo. To further understand the underlying mechanisms through which CTHRC1 promotes tumour invasion and metastasis, we performed ELISAs to detect CTHRC1 in 92 clinical NSCLC serum samples. Several MMPs correlated with CTHRC1 expression. Among them, MMP7 and MMP9, were the two MMPs that were highly correlated with CTHRC1 (Fig. 3a, c, Additional file 1: Figure S3A). To further study the correlation between CTHRC1 and MMP7 as well as MMP9 in fresh primary tumour tissues, we measured the expression of CTHRC1 and MMPs by performing reverse dot blot hybridization. Consistent with the ELISA data, CTHRC1 expression was significantly correlated with MMP7 and MMP9 expression in primary tumour tissues (Fig. 3b, d, Additional file 1: Figure S3B). The correlation between CTHRC1 and MMP7 and MMP9 expression was further confirmed by IHC results obtained from 230 clinical NSCLC tumour samples. Among 127 cases with CTHRC1 high expression, 114 and 115 cases exhibited high MMP7 and MMP9 expression, respectively. Simultaneously, 92 and 85 cases exhibited low MMP7 and MMP9 expression, respectively, out of a total of 103 cases with low CTHRC1 expression (Additional file 1: Figure S3C, 3D).

We next examined the CTHRC1-mediated regulation of MMP7 and MMP9 in in vitro experiments. Both MMP7 and MMP9 were upregulated when CTHRC1 was overexpressed in NSCLC cells. In contrast, CTHRC1 knockdown in NCI-H1975 and NCI-H2122 cells downregulated MMP7 and MMP9 expression at the protein level, as measured by western blotting (Fig. 3e-h).

### NSCLC invasion and migration mediated by CTHRC1 are MMP7- and MMP9-dependent

**Fig. 4:**
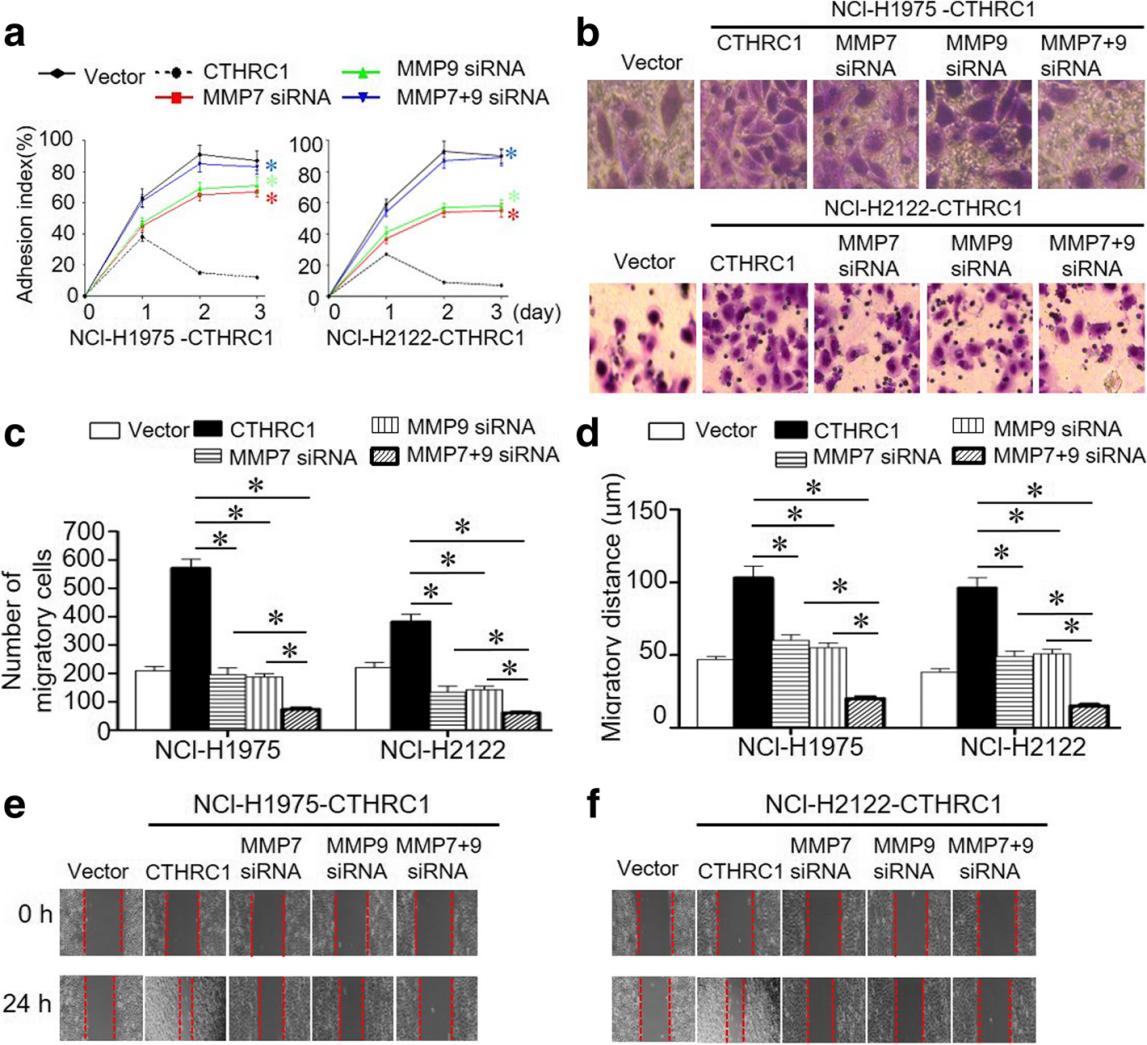
The regulation of CTHRC1 on adhesion, migration, invasion and metastasis of tumor cells was mediated by MMP7 and MMP9. **a** Tumour cell adhesion ability decreased in CTHRC1-overexpressing cells. This decreased adhesion ability was elevated when either MMP7 or MMP9 was knocked down. **b, c** HE staining of cells invading through the Transwell gel demonstrated increased tumour cell invasion accompanying the ectopic overexpression of CTHRC1, which was inhibited by knocking down either MMP7 or MMP9. **d-f** A wound assay showing increased migration distance accompanying CTHRC1 overexpression. Knocking down either MMP7 or MMP9 inhibited the increased migration distance mediated by CTHRC1. *p < 0.05 and **p < 0.01

Based on our data, MMP7 and MMP9 were modulated by CTHRC1 in NSCLC cells. We then sought to determine whether NSCLC invasion and metastasis mediated by CTHRC1 requires MMP7 or MMP9. siRNA was applied to NCI-H1975-CTHRC1 and NCI-H2122-CTHRC1 cells to achieve MMP7 or MMP9 downregulation. Knockdown efficiency was confirmed by RT-PCR, the results of which are shown as a bar graph (Additional file 1: Figure S4A, 4B). CTHRC1 overexpression decreased NSCLC cell adhesion ability, and the adhesion index was significantly increased by either MMP7 or MMP9 downregulation (Fig. 4a). According to our Transwell results, CTHRC1 overexpression increased tumour invasion. However, when MMP7 or MMP9 was knocked down in CTHRC1-overexpressing cells, respectively, they exhibited significant difference in invasion ability compared to those CTHRC1-overexpressing cells without MMP7 or MMP9 knock-down (Fig. 4b, c). Additionally, significant difference was observed in a scratch assay between CTHRC1-overexpressing cells with and without MMP7 or MMP9 knocked-down, respectively (Fig. 4d-f). More significant difference was observed in a adhesion, Transwell and scratch assay in CTHRC1-overexpressing cells with both MMP7 and MMP9 knocked-down, compared with those with MMP7 or MMP9 knocked-down, respectively. Furthermore, we did not observe the changed expression of MMP7 or MMP9 when MMP9 or MMP7 was knocked down (Additional file 1: Figure S4C, 4D).

### CTHRC1 enhances MMP7 promoter activity through AP-1/c-Jun pathway

**Fig. 5:**
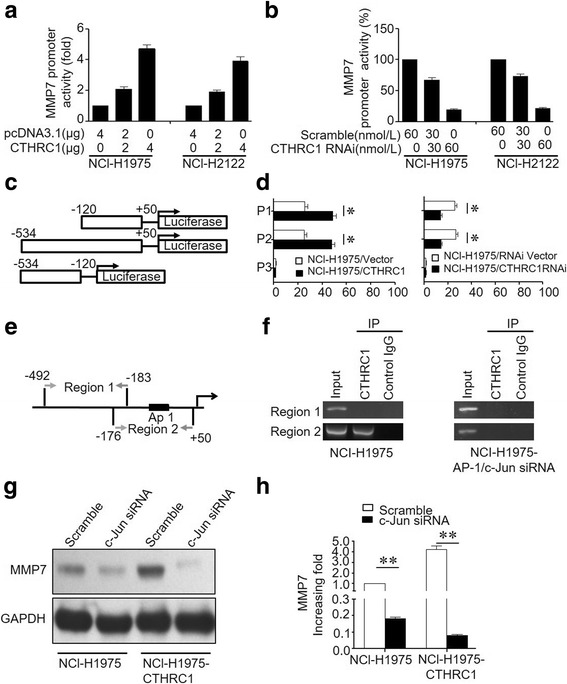
CTHRC1 transcriptionally modulates MMP7 expression through c-JUN. **a** CTHRC1 overexpression increased MMP7 promoter activity. **b** MMP7 promoter activity was inhibited when CTHRC1 was downregulated. **c** The MMP7 promoter region was cloned as three fragments (P1 to P3). **d** Transactivating activity of CTHRC1 on serial MMP7 promoter fragments, as indicated in NCI-H1975 cells. CTHRC1 overexpression enhanced promoter activity in P1 and P2. CTHRC1 knockdown weakened promoter activity in P1 and P2. **e** Schematic illustration showing the PCR-amplified fragments of the MMP7 promoter. **f** Regions of the MMP7 promoter that were physically associated with CTHRC1 were analysed in a ChIP assay. IgG was used as a negative control. PCR amplification indicated the binding efficiency to region 2 was significantly decreased in NCI-H1975-AP-1/c-Jun siRNA cells. **g** CTHRC1 overexpression upregulated the expression of MMP7, and the upregulation of MMP7 was abolished when c-JUN was knocked down as measured by western blot. Representative bands are shown. **h** The western blot results were semi-quantified and are shown in bar graph form. Experiments had been repeated three times. *p < 0.05, **p < 0.01

To further characterize how CTHRC1 upregulates MMP7 expression, a luciferase reporter gene assay was carried out after the nuclear localization of CTHRC1 was confirmed by western blot in NCl-H1975 and NCl-H2122 cells (Additional file 1: Figure S5). NCI-H1975 and NCI-H2122 cells were co-transfected with the MMP7 promoter-luciferase construct pGL3 together with pcDNA3.1-CTHRC1 or a control vector, or CTHRC1 siRNA or scrambled RNA. As shown in Fig. 5a, MMP7 promoter-mediated luciferase activity was enhanced by the co-transfection of pcDNA3.1-CTHRC1 in a dose-dependent manner. In contrast, luciferase activity driven by the MMP7 promoter declined in both NCI-H1975 and NCI-H2122 cells transfected with CTHRC1-RNAi (Fig. 5b). Additionally, serial nucleotide sequences, specifically − 120 to + 50 (P1) and − 534 to + 50 (P2) in the MMP7 promoter region, were cloned into pGL3 (Fig. 5c). Compared to vector-treated cells, the corresponding effects of these serial nucleotide sequences on luciferase activity were significantly increased by the ectopic overexpression of CTHRC1 or decreased by CTHRC1 knockdown. However, the MMP7 promoter fragment spanning − 534 to − 120 (P3) on the luciferase activity appeared to exert no differential effects when combined with either CTHRC1 overexpression or knockdown, compared to matched controls (Fig. 5d, Additional file 1: Figure S6A). Thus, CTHRC1 expression may be involved in the regulation of MMP7 promoter activity through the P1 and P2 regions (nucleotides − 120 to + 50 and − 534 to + 50).

According to the PCR results obtained from the ChIP assay, the physical interaction site between CTHRC1 and the MMP7 gene may be located in region 2 (nucleotides − 176 to + 50) of the MMP7 promoter (Fig. 5e, f). Because CTHRC1 itself does not contain DNA-binding sites, thus, there may be other transcription factors that cooperate with CTHRC1 to enhance promoter activity. Next, the MMP7 promoter region was screened for transcriptional binding sites using prediction tools. A potential binding site in region 2, specifically nucleotides − 176 to + 50 within the MMP7 promoter, was identified as an activator protein (AP)-1–binding element (ABE), as indicated in Fig. 5e. Furthermore, silencing AP-1/c-Jun using siRNA significantly inhibited the binding efficiency of CTHRC1 to the MMP7 promoter (Fig. 5f), implying that AP-1 served as a “bridge protein” between CTHRC1 and MMP7 promoter. Moreover, according to our western blot and RT-PCR results, MMP7 expression was significantly increased in NCI-H1975-CTHRC1 cells compared to NCI-H1975 cells. However, the increased expression of MMP7 in NCI-H1975-CTHRC1 cells was abolished when c-Jun siRNA was introduced (Fig. 5g, h), and MMP7 concentrations in the culture supernatants, as measured by ELISA, significantly decreased when c-Jun was depleted (Additional file 1: Figure S7A, C). Taken together, these findings confirm the involvement of the AP-1 pathway in the CTHRC1-mediated regulation of MMP7.

### CTHRC1 enhances MMP9 promoter activity through the NF-κB and AP-1 pathways

**Fig. 6:**
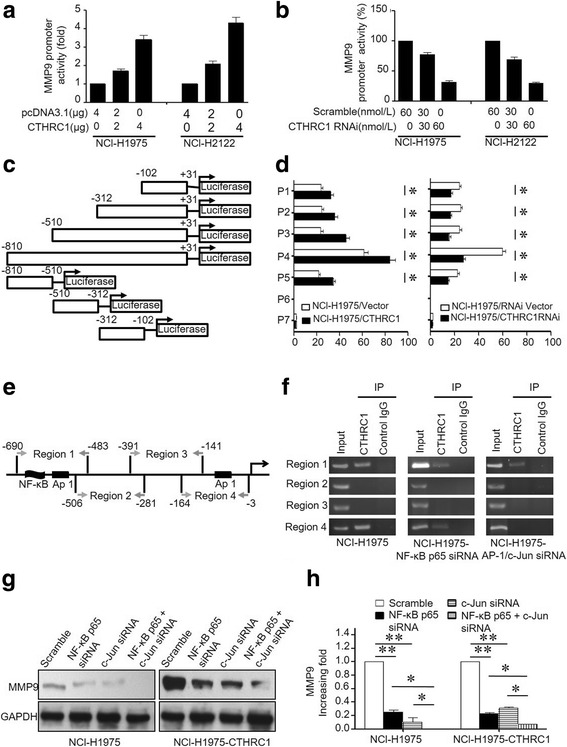
CTHRC1 transcriptionally modulates MMP9 expression through c-JUN and NF-κB signals. **a** CTHRC1 overexpression increased MMP9 promoter activity. **b** MMP9 promoter activity was inhibited when CTHRC1 was downregulated. **c** The promoter region was cloned as seven fragments (P1 to P7). **d** Transactivating activity of CTHRC1 on serial MMP9 promoter fragments as indicated in NCI-H1975 cells. CTHRC1 overexpression enhanced the promoter activity in P1–5, while CTHRC1 knockdown weakened promoter activity in P1–5. **e** Schematic illustration showing the PCR-amplified fragments of the MMP9 promoter. **f** Regions of the MMP9 promoter that were physically associated with CTHRC1 were analysed in a ChIP assay. IgG was used as a negative control. PCR amplification indicated the binding efficiencies to region 1 and region 4 were decreased in NCI-H1975-AP-1/c-Jun siRNA cells, and binding efficiency to region 1 was significantly decreased in NCI-H1975-NF-κB siRNA cells. **g** Western blotting revealed upregulated MMP9 expression accompanying the ectopic overexpression of CTHRC1; this MMP9 upregulation was abolished when either NF-κB or c-JUN was knocked down. Knockdown of NF-κB and c-JUN together further decreased MMP9 expression. Representative bands are shown. **h** The western blot results were semi-quantified and are shown in bar graph form. Experiments had been repeated 3 times. *p < 0.05, **p < 0.01

Employing the same assay described above, we observed the dose-dependent effects of both CTHRC1 and CTHRC-RNAi on MMP9 promoter luciferase activity, similar to those observed for the MMP7 promoter (Fig. 6a, b). We also tested the luciferase activity driven by serial nucleotide fragments within the MMP9 promoter region, specifically − 102 to + 31 (P1), − 312 to + 31 (P2), − 510 to + 31 (P3), − 810 to + 31 (P4), − 810 to − 510 (P5), − 510 to − 312 (P6) and − 312 to − 102 (P7), as shown in Fig. 6c and d. CTHRC1 overexpression increased MMP9 promoter activity but decreased when CTHRC1 was knocked down (Fig. 6c, d and Additional file 1: Figure S6B). Potential binding sites within the MMP9 promoter region were identified in region 1 for NF-κB and Ap-1 (nucleotides − 690 to − 483) and within region 4 for Ap-1 (nucleotides − 164 to − 3) (Fig. 6e). Additionally, the binding efficiencies of CTHRC1 to MMP9 promoter regions 1 and 4 were reduced by the application of AP-1/c-Jun siRNA. NF-κB p65 siRNA significantly suppressed the binding capacity of CTHRC1 for region 1 within the MMP9 promoter (Fig. 6f). Moreover, western blot and ELISA results further verified the upregulation of MMP9 expression by CTHRC1 through NF-κB and AP-1 pathways at both the protein and mRNA levels (Fig. 6g, h, Additional file 1: Figure S7B, D).

### Increased expression of CTHRC1 was correlated with CTC and predicts progression and poor prognosis of NSCLC

**Fig. 7:**
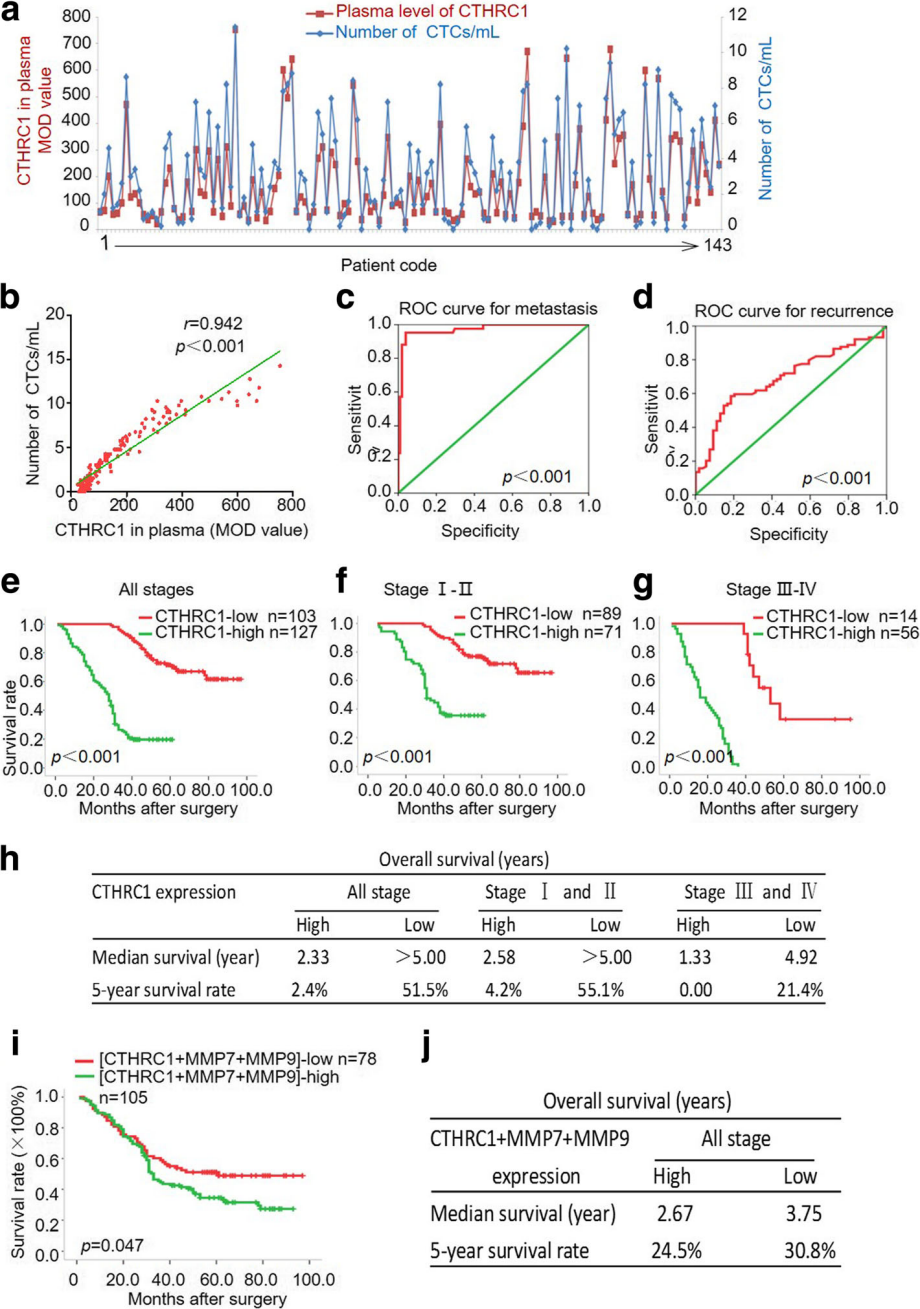
Clinical relevance of CTHRC1 expression in NSCLC patients. **a, b** Circulating tumour cells (CTC) were detected using the NanoVelcro system. The CTHRC1 concentration positively correlated with the number of CTCs. **c, d** The cut-off value for CTHRC1 had optimal sensitivity and specificity for metastasis, with an area under the curve of 0.97 (95% CI: 0.941–1.000; *p* < 0.001), and optimal sensitivity and specificity for recurrence, with an area under the curve of 0.691 (95% CI: 0.604–0.788; *p* < 0.001). **e** IHC data indicating the post-surgery survival rate was significantly lower in the group of CTHRC1-high patients. **f, g** CTHRC1-low patients had much higher post-surgery survival rates, at both the early and late stages. **h** Summary of overall survival with high or low CTHRC1 expression. **i** Compared to low CTHRC1, MMP7 and MMP9 expression, the combined high expression of CTHRC1, MMP7 and MMP9 was associated with a significantly lower post-surgery survival rate. **j** Summary of overall survival with high or low combinatorial CTHRC1, MMP7 and MMP9 expression

Further investigation is required to understand the relationship between CTHRC1 and clinical metastasis in NSCLC. Tumour cells are detectable in the circulating blood of cancer patients but not in that of healthy individuals or patients with non-malignant diseases. The increased numbers of CTCs indicate a higher chance for tumour metastasis. In the present study, we detected CTC numbers using a NanoVelcro system and measured serum CTHRC1 concentrations by ELISA (*n* = 143) in the same NSCLC patients. Compared to normal controls, serum levels of CTHRC1 were significantly higher in NSCLC patients (*n* = 40). CTHRC1 concentrations in NSCLC patients correlated with metastasis. Advanced disease stages characterized by local invasion or distant metastasis exhibited much higher serum CTHRC1 levels (Additional file 1: Figure S8A-C). Furthermore, CTHRC1 concentration significantly correlated with CTC counts (Fig. 7a, b). Additionally, the CTHRC1 cut-off had optimal sensitivity and specificity for metastasis with an area under the curve of 0.97 (95% CI: 0.941–1.000; *p* < 0.001). The CTHRC1 cut-off also had optimal sensitivity and specificity for recurrence with an area under the curve of 0.691 (95% CI: 0.604–0.788; *p* < 0.001) (Fig. 7c, d).

Given the role of CTHRC1 in the tumor invasion and metastasis, we made further efforts to identify the clinical significance of CTHRC1 for prognosis prediction in NSCLC patients. Patients with high CTHRC1 expression had significant lower 5-year survival rate (2.4%) than those with low CTHRC1 expression (51.5%) (Fig. 7e). Moreover, early stage (stages I and II) and late stage (stages III and IV) patients with high CTHRC1 expression had shorter survival durations than those with low CTHRC1 expression (Fig. 7f-h). In the early and advanced stages, 5-year survival rates for low CTHRC1 expression were 55.1% and 21.4%, respectively. All the advanced stage patients with high CTHRC1 expression died within 5 years.

In a univariate analysis, CTHRC1 levels were correlated with overall survival (Additional file 1: Table S6). Further, a multivariate analysis performed using the COX proportional hazard regression model indicated CTHRC1 overexpression was an independent prognosis-related marker for NSCLC (Additional file 1: Table S7). We then evaluated how predictive prognostic power was related to the combined expression of CTHRC1, MMP7 and MMP9 in NSCLC patients. Compared to patients with a low expression pattern, patients with a high expression pattern for CTHRC1, MMP7 and MMP9 appeared to have poorer survival (Fig. 7i, j).

## Discussion

Cancer invasion and metastasis are among the biological hallmarks acquired during the multistep development of human tumours. Underlying these hallmarks is genome instability, which leads to the genetic diversity that eventually expedites their acquisition [30, 31]. In this report, CTHRC1 upregulation was associated with lymphatic metastasis, distant metastasis, and MMP7 and MMP9 overexpression in NSCLC patients, indicating CTHRC1 plays a critical role in promoting cancer invasiveness. Consistent with the above findings, we identified MMP7 and MMP9 as substrates of CTHRC1 and revealed an unknown function of CTHRC1 in promoting strong cell invasion in an MMP7- and MMP9-dependent manner through the transcriptional upregulation of MMP7 and MMP9 via interactions with their corresponding promoters.

The most common causes of cancer-associated mortality are the occurrence of local invasion or distant metastasis, rather than the presence of the primary tumours themselves. Among clinically diagnosed NSCLC patients, almost half have confirmed distant metastases [32]. As a candidate tumour marker in this study, CTHRC1 was identified in NSCLC samples by performing quantitative assessments involving 2D-PAGE gels and mass spectrometry in comparison with adjacent non-tumour tissues. Furthermore, elevated preoperative serum CTHRC1 levels were associated with tumour metastasis. According to previous studies [20, 22, 29], and our own [23]. CTHRC1 promotes cancer progression and activates relevant signalling molecules, which urged us to determine whether CTHRC1 plays a similar role in determining the aggressiveness of NSCLC. Thus, we evaluated CTHRC1 functions in lung cancer cell migration and invasion. Consistent with the published effects of CTHRC1 on the invasive phenotype of NSCLC cells, CTHRC1 knockdown greatly decreased cell invasion and inhibited cell migration, whereas endogenous CTHRC1 overexpression significantly increased invasive ability. Additionally, based on the IHC analysis of 230 clinical NSCLC specimens, CTHRC1 overexpression was significantly correlated with clinical stage, indicating CTHRC1 upregulation may facilitate NSCLC metastasis.

Tumour metastasis is initiated by a CTC sub-group that has been observed in patient blood. CTCs are used as a marker to predict disease progression in metastatic patients, including breast, colorectal and prostate cancers. They are involved in metastatic spread to distant organs, leading to the formation of secondary sites of lung cancer [33]. Here, increased plasma levels of CTHRC1 were positively correlated with the presence of CTCs, suggesting plasma CTHRC1 attracts CTCs into circulation. However, the molecular mechanisms underlying tumour metastasis mediated by CTHRC1 require further investigation.

Proteolytic degradation of the stromal ECM is well known for its contribution to malignant invasion and metastasis [34, 35]. MMPs, as a family of zinc-dependent endopeptidases, are involved in degrading ECM and facilitating tumour invasion [11, 36, 37]. As shown in the present study, significantly higher expression of CTHRC1, MMP-7 and MMP-9 was observed in a cohort of NSCLC sera and surgically resected tumour tissues. CTHRC1 was positively correlated with MMP7 and MMP9 expression at both the protein and mRNA levels. MMP regulation, generally by hormones and cytokines, occurs primarily at the transcription level [38, 39]. MMPs are also regulated via the initiation of pro-MMP cleavage and proteolytic activity inhibition by specific inhibitors [40–42]. Our observations here indicate MMP7 and MMP9 may serve as the potential target genes of CTHRC1 in NSCLC, which may explain why CTHRC1 enhances NSCLC progression in vivo. However, it remains unclear whether the involvement of MMP7 and MMP9 in NSCLC progression is mechanistically regulated by CTHRC1.

CTHRC1, generally recognized as a secreted protein [19], was firstly confirmed in the nuclear localization of NSCLC cells. This builds the foundation for us to deeply explore the regulation mechanisms of CTHRC1 on MMP7 and MMP9. MMP7 and MMP9 expression is generally regulated via promoter binding sites for multiple transcription activators, such as AP-1 and β-catenin/TCF4 for MMP7 [43, 44] and AP-1, AP-2, NF-κB, and SP-1 for MMP9 [45, 46]. Based on previous findings from a luciferase-based ChIP assay, CTHRC1 binds to the MMP7 promoter (− 120 to + 50 bp) and MMP9 promoter (− 102 to + 31 bp and − 810 to − 510 bp). However, CTHRC1 does not contain DNA-binding sites [22].

Thus, CTHRC1 may cooperate with other transcription factors to bind to the promoters of downstream genes and initiate to their transcriptional activation. Sequence analysis revealed a potential binding site for AP-1 in the MMP7 promoter between nucleotide − 176 to + 50 bp. Two binding sites for NF-κB and AP-1 are also present in the MMP9 promoter between nucleotides − 164 to − 3 bp and − 690 to − 506 bp, respectively. Furthermore, AP-1/c-Jun depletion decreased the binding efficiency of CTHRC1 to the MMP7 promoter. Simultaneously, the knockdown of both NF-κB and AP-1/c-Jun suppressed CTHRC1 binding to the MMP9 promoter. In agreement with our hypothesis, c-Jun depletion downregulated MMP7 expression, and knockdown of either NF-κB or c-Jun decreased MMP9 expression. However, whether the invasion mediated by CTHRC1 depends on MMP7 and MMP9 remains unclear.

The metalloproteinases MMP7 and MMP9 are overexpressed in NSCLC and other types of cancers and are strongly associated with poor prognosis [16, 47, 48]. An inhibitor of MMPs, including MMP7 and MMP9, was shown to suppress tumour metastasis [49]. Additionally, the application of MMP7 to colon cancer-bearing nude mice enhanced tumour metastasis [50] and MMP9-deficient mice were protected against tumour metastasis [14]. Consistent with these data, the enhanced tumour cell migration and invasion mediated by CTHRC1 overexpression was eradicated when either MMP7 or MMP9 was knocked down. Based on these data, CTHRC1-mediated invasion is MMP7- and MMP9-dependent.

In conclusion, our current systematic study identified CTHRC1 as a invasion-driving gene that promotes NSCLC progression by activating c-Jun/MMP7, c-Jun/MMP9 and NF-κB/MMP9 signalling; thus, therapeutic targeting of CTHRC1 may be a promising strategy to enhance the therapeutic effects of anticancer drugs against NSCLC. Additionally, CTHRC1 may serve as a sensitive predictor of the low 5-year overall survival in NSCLC and as an effective biomarker for evaluating the poor clinicopathological characteristics of NSCLC.

## Conclusions

CTHRC1 promotes NSCLC invasion by upregulating MMP7 and MMP9. Targeting CTHRC1 may be beneficial for inhibiting NSCLC progression.

## Additional file

Additional file 1: Supplementary data. (DOCX 5937 kb)

## Abbreviations

ANTs: Non-tumour tissues
CTC: Circulating tumour cell
CTHRC1: Collagen triple helix repeat containing 1
ECM: Extracellular matrix
MMP-7: Matrix metalloproteinase 7
MMP-9: Matrix metalloproteinase 9
NSCLC: Non-small cell lung cancer
