# Supplementary material for: CTHRC1 induces non-small cell lung cancer (NSCLC) invasion through upregulating MMP-7/MMP-9
Source: BMC Cancer. 2018 Apr 10;18:400. doi: 10.1186/s12885-018-4317-6 (PMC5891957; doi:10.1186/s12885-018-4317-6)
Supplement: Supplementary file 1 — Supplementary data. (DOCX 5937 kb) [file 12885_2018_4317_MOESM1_ESM.docx]

**Additional file 1**

**Methods and materials**

**Reagents**

The overexpression plasmid pcDNA3.1, used to induce CTHRC1, MMP7 and MMP9 overexpression, and GV248-RNAi, targeting CTHRC1, MMP7 and MMP9 stable transfection, were acquired from Jikai (Shanghai, China). NF-κB small interfering RNA (siRNA) and c-Jun-siRNA were purchased from RiboBio Inc. (Guangzhou, China). Lipofectamine 2000 was purchased from Invitrogen (CA, USA). Antibodies against CTHRC1, NF-κB, c-Jun, MMP-7, MMP-9, TTF-1, CEA, CK-7, p63, Histone 2 and GAPDH were purchased from Abcam (Cambridge, UK). Horseradish peroxidase (HRP)-conjugated anti-rabbit or anti-mouse IgG was purchased from Cell Signaling Technology (MA, USA). FITC-anti-cytokeratin and PE-anti-CD45 antibodies were purchased from BioLegend (CA, USA).

**Cell lines and culture conditions**

Lung cancer cell lines, specifically A-549, NCI-H1650, NCI-H1975, NCI-H2228, HC-C827, NCI-H23, NCI-H820, NCI-H1993, NCI-H2122, and NCI-H522, were all purchased from the American Type Culture Collection (ATCC). Cells were maintained in Dulbecco’s Modified Eagle’s medium (DMEM, Invitrogen, USA), supplemented with 10% foetal bovine serum (HyClone, USA). Primary normal lung epithelial cells (BEAS-2B) were cultured in a keratinocyte serum-free medium (Invitrogen, Carlsbad, CA) supplemented with epidermal growth factor (EGF) (Invitrogen).

**Plasmids, retroviral infection and transfection**

CTHRC1, MMP7 and MMP9 constructs were generated by sub-cloning PCR-amplified full-length human CTHRC1, MMP7 or MMP9 cDNA into pcDNA3.1. To deplete CTHRC1, MMP7 and MMP9, siRNA sequences were cloned into GV248 to generate GV248-RNAi(s) targeting CTHRC1, MMP7 and MMP9. Different regions of the human MMP7 and MMP9 promoters, including fragments covering nucleotides -120 to +50, -534 to +50, -534 to -120, -102 to +31, -312 to +31, -510 to +31, -810 to +31, -810 to -510, -510 to -312, and -312 to -102 (relative to the transcription initiation site), were generated via PCR amplification from NCI-H1975 and cloned into the KpnI/HindIII sites of the pGL3-basic luciferase reporter plasmid (Promega, Madison, WI). siRNA duplexes were synthesized and purified by RiboBio Inc. (Guangzhou, China). siRNAs transfection was carried out using Lipofectamine 2000 reagent (Invitrogen Co., Carlsbad, CA) according to the manufacturer’s instructions. Stable cell lines expressing CTHRC1 or CTHRC1 RNAi(s) were selected via treatment with 0.5 μg/ml puromycin for 10 days beginning 48 h after infection. Following selection, lung cancer cell lysates prepared from the pooled cell populations in sampling buffer were fractionated by sodium dodecyl sulphate-polyacrylamide gel electrophoresis (SDS–PAGE) to detect protein levels via western blotting. siRNA sequences are listed in Supplementary Table 3.

**RT-PCR**

Total RNA was extracted using an RNAeasy Kit (Qiagen, USA) according to the manufacturer’s instructions. cDNA was synthesized using recombinant Taq DNA polymerase (Fermentas, USA). Quantitative real-time PCR was carried out with SYBR Green I (Roche, USA). Amplification was performed under the following conditions: preheating at 95°C for 10 min, denaturing at 95°C for 15 s, and annealing and extension at 65°C for 45 s for a total of 35 cycles. GAPDH was used as the internal control. The primers used in this study are listed in Supplementary Table 4.

**Adhesion assay**

Cell adhesion experiments were performed to test the adhesive capacities of NCI-H1975 and NCI-H2122 cells. Ninety-six-well plates were coated with fibronectin (10 μg/ml) in PBS for 2 h at 37°C and then blocked with 1% bovine serum albumin (BSA). Cells were collected via centrifugation and suspension (using non-serum RPMI 1640 medium containing 0.1% BSA and 0.1% glucose). In triplicate, 75 × 10^4^ cells/ml in a volume of 100 μl were plated and incubated for the indicated periods of time (24 h, 48 h and 72 h) at 37°C. Careful PBS washes were performed to remove non-adherent cells. The adherent cells were examined under an IX71 inverted microscope (Olympus Corp, Tokyo, Japan).

**Scratch assay**

The lung cancer cell lines NCI-H1975 and NCI-H2122 were grown to 95% confluence on tissue culture dishes. Single scrapes were made in the confluent monolayers with sterile pipette tips. The dishes were washed with PBS to remove cell debris and non-adherent cells. Cells were cultured in serum-free media. Serial photographs of identical scraped sections were taken every 6 h for a total of 24 h. The distances that cells migrated over the margins of the scrapes were measured after 24 h.

**Transwell**

Cell migration assays were performed in triplicate using 8-μm pore filters with 6.5-mm diameter chambers in 24-well plates (Costar, Boston, MA). Then, 50 μl of fibronectin (100 μg/ml) was coated on the lower filter surfaces. The filters were subsequently air-dried. The lung cancer cell lines NCI-H1975 and NCI-H2122 were suspended at 2 × 10^5^ cells/ml in serum-free media. Cell suspensions in a volume of 200 μl were loaded in the upper chambers. Serum-free media was added in the lower chambers. The chambers were then incubated at 37°C in a humidified atmosphere containing 5% CO_2_ for 12 h. After incubation, the filters were fixed in 4% paraformaldehyde for 10 min at room temperature, and HE staining was performed. Non-migrated cells on the upper filter surfaces were removed with cotton swabs. Numbers of migrated cells were counted in five high-power (400×) fields per filter using a microscope (Olympus, Japan).

**Table S1. Correlation between CTHRC1 expression and clinicopathologic characteristics of NSCLC**

| **Clinicopathologic characteristics** | **CTHRC1 expression** | |  | ***p*** | **MMP7 expression** | |  |  | **MMP9 expression** | |  |  |
| --- | --- | --- | --- | --- | --- | --- | --- | --- | --- | --- | --- | --- |
|  | **High** | **Low** |  |  | **High** | **Low** |  | ***p*** | **High** | **Low** |  | ***p*** |
| **Normal lung tissues** | 11 | 93 |  | <0.001 | 8 | 96 |  | <0.001 | 10 | 94 |  | <0.001 |
| **NSCLC tissues** | 127 | 103 |  |  | 125 | 105 |  |  | 133 | 97 |  |  |
| **Age(years)** |  |  |  | 0.621 |  |  |  | 0.951 |  |  |  | 0.911 |
| ≤50 | 28 | 25 |  |  | 29 | 24 |  |  | 31 | 22 |  |  |
| >50 | 99 | 78 |  |  | 96 | 81 |  |  | 102 | 75 |  |  |
| **Tumor size** |  |  |  | 0.006 |  |  |  | 0.086 |  |  |  | <0.001 |
| ≤3 cm | 46 | 56 |  |  | 49 | 53 |  |  | 44 | 58 |  |  |
| >3 cm | 81 | 47 |  |  | 76 | 52 |  |  | 89 | 39 |  |  |
| **Gender** |  |  |  | 0.041 |  |  |  | 0.296 |  |  |  | 0.211 |
| Male | 83 | 80 |  |  | 85 | 78 |  |  | 90 | 73 |  |  |
| Female | 44 | 23 |  |  | 40 | 27 |  |  | 43 | 24 |  |  |
| **Smoking** |  |  |  | <0.001 |  |  |  | 0.026 |  |  |  | 0.008 |
| Yes | 41 | 59 |  |  | 46 | 54 |  |  | 48 | 52 |  |  |
| No | 86 | 44 |  |  | 79 | 51 |  |  | 85 | 45 |  |  |
| **Clinical stage** |  |  |  | <0.001 |  |  |  | <0.001 |  |  |  | <0.001 |
| I | 43 | 60 |  |  | 42 | 61 |  |  | 39 | 64 |  |  |
| II | 28 | 29 |  |  | 27 | 30 |  |  | 33 | 24 |  |  |
| III | 35 | 12 |  |  | 33 | 14 |  |  | 39 | 8 |  |  |
| IV | 21 | 2 |  |  | 23 | 0 |  |  | 22 | 1 |  |  |
| **T Classification** |  |  |  | <0.997 |  |  |  | 0.978 |  |  |  | 0.822 |
| T1 | 47 | 39 |  |  | 48 | 38 |  |  | 53 | 33 |  |  |
| T2 | 54 | 44 |  |  | 52 | 46 |  |  | 55 | 43 |  |  |
| T3 | 14 | 11 |  |  | 14 | 11 |  |  | 14 | 11 |  |  |
| T4 | 12 | 9 |  |  | 11 | 10 |  |  | 11 | 10 |  |  |
| **N Classification** |  |  |  | <0.001 |  |  |  | <0.001 |  |  |  | <0.001 |
| N0 | 60 | 72 |  |  | 56 | 76 |  |  | 58 | 74 |  |  |
| N1 | 20 | 17 |  |  | 21 | 16 |  |  | 21 | 16 |  |  |
| N2 | 29 | 13 |  |  | 29 | 13 |  |  | 36 | 6 |  |  |
| N3 | 18 | 1 |  |  | 19 | 0 |  |  | 18 | 1 |  |  |
| **Lymph node metastasis** |  |  |  | 0.001 |  |  |  | <0.001 |  |  |  | <0.001 |
| N0 | 60 | 72 |  |  | 56 | 76 |  |  | 58 | 74 |  |  |
| N1-3 | 67 | 31 |  |  | 69 | 29 |  |  | 75 | 23 |  |  |
| **M Classification** |  |  |  | <0.001 |  |  |  | <0.001 |  |  |  | <0.001 |
| M0 | 106 | 101 |  |  | 102 | 105 |  |  | 111 | 96 |  |  |
| M1 | 21 | 2 |  |  | 23 | 0 |  |  | 22 | 1 |  |  |
| **EGFR mutation** |  |  |  | 0.067 |  |  |  | 0.995 |  |  |  | 0.005 |
| Positive | 50 | 53 |  |  | 56 | 47 |  |  | 49 | 54 |  |  |
| Negative | 77 | 50 |  |  | 69 | 58 |  |  | 84 | 43 |  |  |
| **K-ras mutation** |  |  |  | 0.229 |  |  |  | 0.762 |  |  |  | 0.926 |
| Positive | 13 | 16 |  |  | 15 | 14 |  |  | 17 | 12 |  |  |
| Negative | 114 | 87 |  |  | 110 | 91 |  |  | 116 | 85 |  |  |
| **ALK rearrangement** |  |  |  | 0.813 |  |  |  | 0.959 |  |  |  | 0.632 |
| Positive | 10 | 9 |  |  | 11 | 8 |  |  | 10 | 9 |  |  |
| Negative | 117 | 94 |  |  | 114 | 97 |  |  | 123 | 88 |  |  |

**Table S2. Sequences for reverse dot blot in this study**

|  | Gene name | cDNA sequence |
| --- | --- | --- |
| 1 | CTHRC1 | CCATGCGACCCCAGGGC |
| 2 | MMP1 | ATGCACAGCTTTCCTCCACT |
| 3 | MMP2 | ATGGAGGCGCTAATGGC |
| 4 | MMP3 | ATGAAGAGTCTTCCAATCCTAC |
| 5 | MMP7 | ATGCGACTCACCGTG |
| 6 | MMP8 | ATGTTCTCCCTGAAGACGCT |
| 7 | MMP9 | ATGAGCCTCTGGCAGCCCCT |
| 8 | MMP10 | ATGATGCATCTTGCATTCCT |
| 9 | MMP11 | ATGGCTCCGGCCGCCT |
| 10 | MMP12 | ATGAAGTTTCTTCTAATACTGCTCC |
| 11 | MMP13 | ATGCATCCAGGGGTCC |
| 12 | MMP14 | ATGTCTCCCGCCCCAAG |
| 13 | MMP15 | ATGGGCAGCGACCCGAGC |
| 14 | MMP16 | ATGATCTTACTCACATTCAGCACTG |
| 15 | MMP17 | ATGCGGCGCCGCGCAG |
| 16 | MMP19 | ATGAACTGCCAGCAGCTGTG |
| 17 | MMP20 | ATGAAGGTGCTCCCTGCAT |
| 18 | MMP21 | ATGCTCGCCGCCTCCATCTTC |
| 19 | MMP23 | ATGGGCTGCCGGGCCTGTC |
| 20 | MMP24 | ATGCCGAGGAGCCGGGGC |
| 21 | MMP25 | ATGCGGCTGCGGCTC |
| 22 | MMP26 | ATGCAGCTCGTCATCTTAAGA |
| 23 | GAPDH | ACCATGGGGAAGGTGAAGG |

**Table S3. siRNA sequences used in this study**

| **Gene** | **Sequences (5’-3’)** |
| --- | --- |
| MMP7  MMP9  CTHRC1  c-Jun  NF-κB p65 | GACCTTATGGCTACAGTAA GCTGCAGTTTGATGATGAA  AACAGAAGAAGAAGAACCGGA  GACCTTATGGCTACAGTAA  AACCCCTTCCAAGTTCCTATA |

**Table S4. PCR primers used in this study**

| Gene | Forward primer | Reverse primer |
| --- | --- | --- |
| MMP7  MMP9  CTHRC1 | ATGCGACTCACCGTG  ATGAGCCTCTGGCAGCCCCT  CCATGGAGAAGGCTGGG | CTATTTCTTTCTTGAATTACTT  CTAGTCCTCAGGGCACTGCAGG  CAAAGTTGTCATGGATGACC |

**Table S5. Differential protein expression identified in NSCLC and normal lung tissues**

| No. | Proteins name | Protein AC | pI | MW (Da) | MASCOT  score | Sequence  coverage (%) | Fold change* |
| --- | --- | --- | --- | --- | --- | --- | --- |
| A1  A2  A3  A4  A5  A6  A7  A8  B1  B2  B3  B4  B5  B6  C1  C2  C3  C4  D1  D2  D3  D4  D5  D6  D7  D8  D9  D10  D11  D12  D13  D14  D15  D16 | ALG-2 interacting protein 1  Zyxin  Glycyl-tRNA synthetase  Thioredoxin reductase  Similar to angio-associated, migratory cell protein, partial  Nascent polypeptide-associated complex subunit alpha isoform b  Nicotinamide N-methyltransferase  Chain A, Familial Als Mutant G37r Cuznsod  Vimentin variant, partial  Ubiquilin-1 isoform 2  Galactokinase  Poly(rC)-binding protein 1  TALDO1 protein, partial  Chain A, Crystal Structure of an Rb C-terminal peptide bound to the catalytic subunit of Pp1  SET, partial  Proteasome activator complex subunit 1 isoform 2  Predictive: similar to splicing factor, arginine/serine-rich 3  Chain A, Crystal structure of the human Co-chaperone P23  Protein-glutamine gamma-glutamyltransferase 2 isoform a  BiP protein, partial  Mitochondrial ATP synthase, H+ transporting F1 complex beta subunit, partial  PSMC3  Ubiquitin-conjugating enzyme E2 Z  Heterogeneous nuclear ribonucleoprotein C (C1/C2), isoform CAR_c  TIMM50 protein  Unnamed protein product  Heterogeneous nuclear ribonucleoprotein H3 isoform b  Heterogeneous nuclear ribonucleoprotein H3 isoform a  Inorganic pyrophosphatase  **CTHRC1**  Ubiquitin carboxyl-terminal hydrolase isozyme L1  Calpain small subunit 1 isoform 1  Proteasome subunit beta type-6 isoform 1 precursor  Mitochondrial import receptor subunit TOM22 homolog | gi\|6424942  gi\|4508047  gi\|600727  gi\|1184537  gi\|18044384  gi\|163965364  gi\|5453790  gi\|2982080  gi\|62896523  gi\|16753205  gi\|4503895  gi\|6754994  gi\|48257056  gi\|302566194  gi\|145843637  gi\|30581141  gi\|224084730  gi\|9257073  gi\|39777597  gi\|6470150  gi\|89574029  gi\|48145579  gi\|150417999  gi\|119586801  gi\|14290586  gi\|194381644  gi\|14141159  gi\|14141157  gi\|11056044  gi\|371120669  gi\|21361091  gi\|4502565  gi\|23110925  gi\|9910382 | 6.13  6.22  5.88  6.07  4.31  4.52  5.56  5.87  5.06  5.01  6.04  6.66  6.35  5.04  4.73  6.28  10.12  5.09  5.11  5.23  4.95  5.39  5.37  10.22  8.56  6.02  6.36  6.37  5.54  6.45  5.33  5.05  4.80  4.27 | 96646  62436  78166  55126  46842  23370  30011  16122  53708  59183  42702  37987  37556  35051  26593  28755  14422  15146  78420  71002  48083  45507  38642  19079  39014  32940  35273  36960  33095  25646  25151  28469  25570  15512 | 414  262  86  367  238  235  385  162  239  395  557  255  326  229  336  223  188  222  188  611  654  363  154  367  277  310  360  476  330  419  402  548  454  114 | 20  18  4  16  12  22  25  35  21  20  37  16  31  35  40  32  32  31  18  31  49  29  16  33  21  31  40  47  38  32  64  37  43  33 | 0.33  0.36  0.24  0.38  0.62  0.39  0.19  0.12  3.03  3.36  2.83  1.31  2.07  2.06  0.24  0.37  0.78  0.16  3.74  1.69  2.02  3.13  2.12  1.36  4.38  1.78  2.19  4.17  3.62  12.88  3.52  2.99  2.05  1.24 |

* NSCLC tumour tissues vs. normal lung tissues

**Table S6. Univariate analysis of survival time**

|  | HR | 95% CI for HR | P-value |
| --- | --- | --- | --- |
| **Gender** (Male vs. Female) | 0.71 | (0.50 , 1.02) | 0.062 |
| **Age, years** (>50 vs. ≤50) | 0.90 | (0.59 , 1.35) | 0.605 |
| **Tumor size** (>3 cm vs. ≤3 cm) | 1.53 | (1.08 , 2.17) | 0.016 |
| **Smoking** (No vs. Yes) | 0.73 | (0.52 , 1.03) | 0.073 |
| **Clinical stage** (III+IV vs. I+ II) | 4.61 | (3.25 , 6.54) | <0.001 |
| **Lymph node** (N1-3 vs. N0) | 3.93 | (2.75 , 5.61) | <0.001 |
| **M Classification** (M1 vs. M0) | 5.78 | (3.57 , 9.36) | <0.001 |
| **CTHRC1** (high vs. low) | 7.50 | (4.85 , 11.59) | <0.001 |
| **MMP7** (high vs. low) | 7.38 | (4.83 , 11.28) | <0.001 |
| **MMP9** (high vs. low) | 6.97 | (4.47 , 10.87) | <0.001 |

HR: Hazard ratio

**Table S7. Multivariate analysis of survival time**

|  | B | HR | 95 % CI for HR | | P-value |
| --- | --- | --- | --- | --- | --- |
|  |  |  | Lower | Upper |  |
| **Lymph node** (N1-3 vs. N0) | 1.07 | 2.91 | 1.81 | 4.69 | <0.001 |
| **Clinical stage** (III+IV vs. I+ II) | 0.59 | 1.81 | 1.14 | 2.87 | 0.011 |
| **Tumor size** (>3 cm vs. ≤3 cm) | 0.10 | 1.11 | 1.01 | 1.21 | 0.031 |
| **CTHRC1** (high vs. low) | 1.34 | 3.80 | 2.03 | 7.11 | <0.001 |
| **MMP7** (high vs. low) | 0.93 | 2.54 | 1.37 | 4.69 | 0.003 |
| **MMP9** (high vs. low) | 0.53 | 1.70 | 1.03 | 2.80 | 0.037 |

**Additional file figures and figure legends**


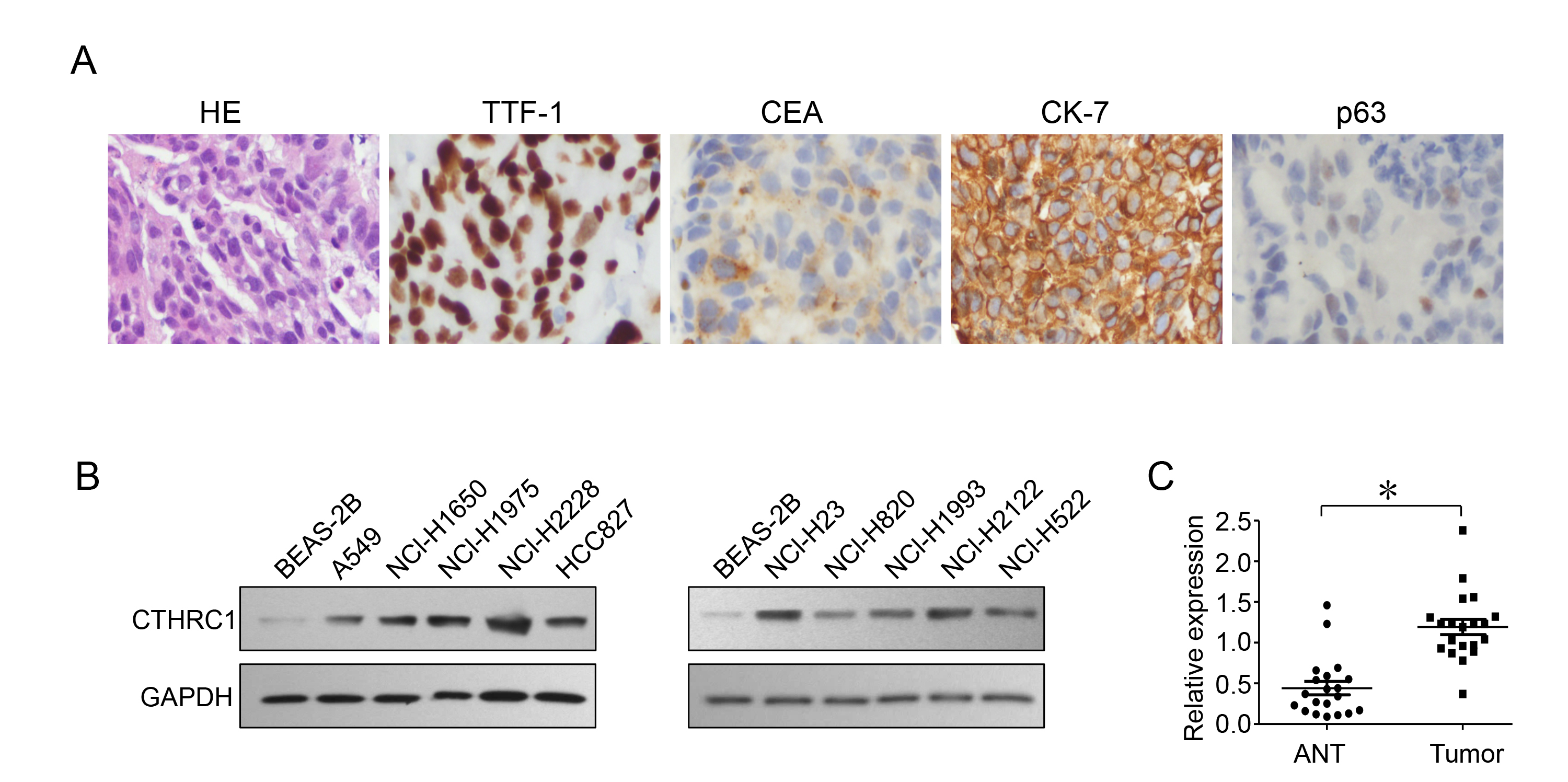


**Figure S1**. **Overexpression of CTHRC1 in NSCLC cell lines**. Clinic NSCLC samples were collected from the pathology sample archive. (**A**) All samples used in this study were stained with HE and antibodies against TTF-1, CEA, CK-7 and p63 as biological markers to confirm NSCLC. (**B**) Western blotting showed the increased CTHRC1 expression in NSCLC cells lines. (C) RT-PCR demonstrated CTHRC1 overexpression in NSCLC clinical tissue samples (n=20). **p*<0.05.


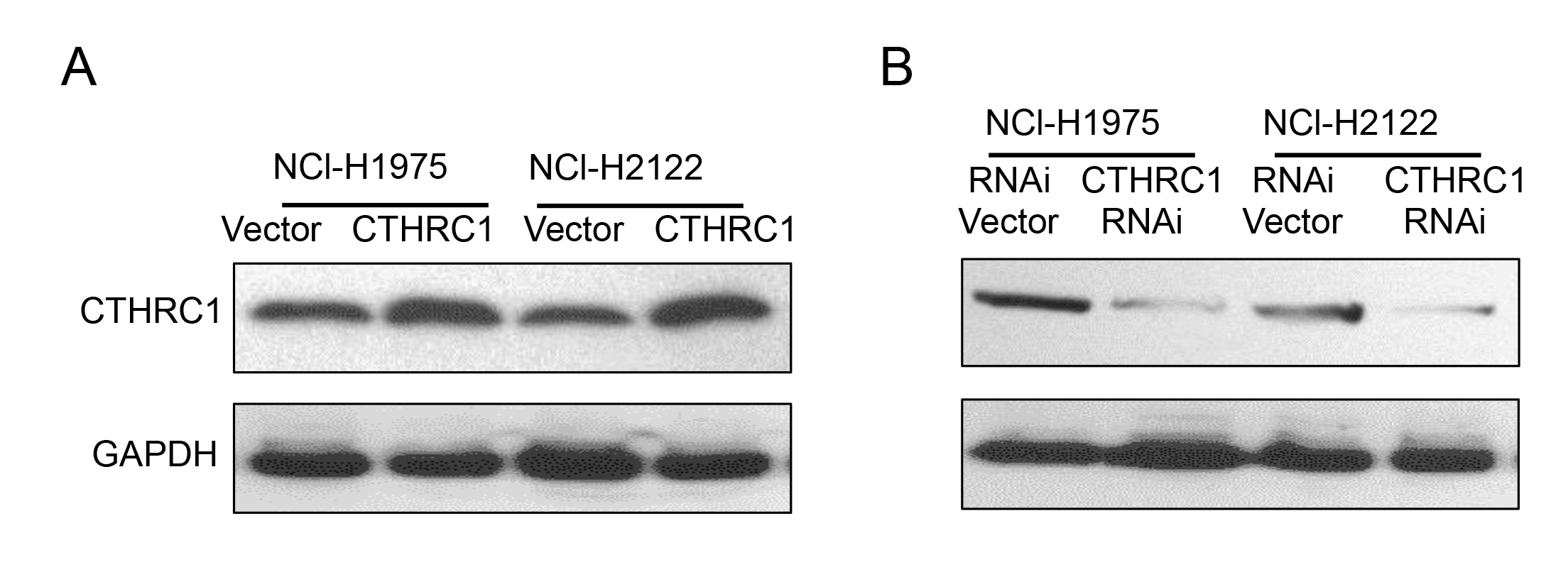


**Figure S2.** Western blotting demonstrated the efficiencies of overexpression or knockdown of CTHRC1 in NCI-H1975 and NCI-H2122 cells.


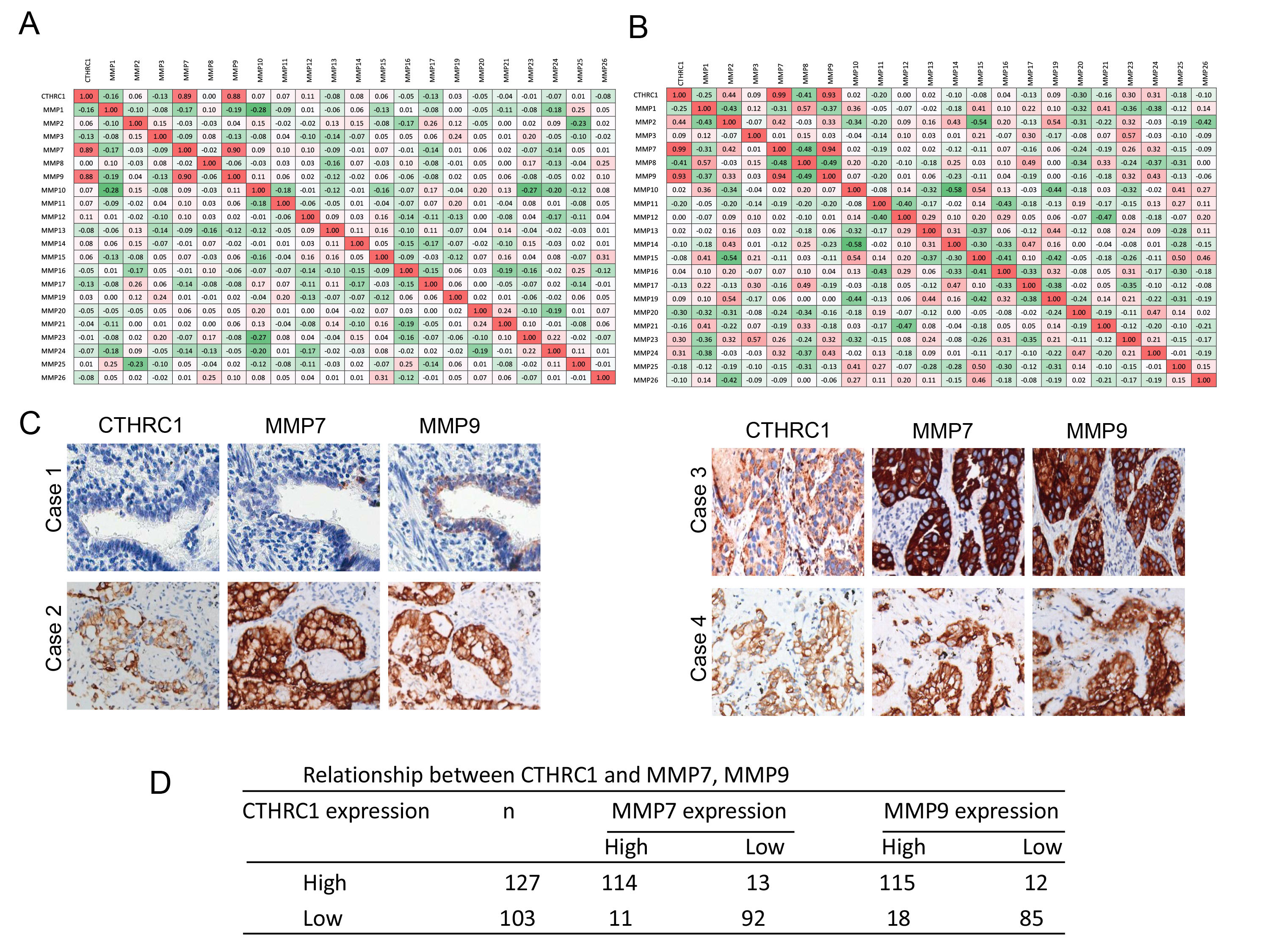


**Figure S3**. **Positive correlation between CTHRC1 and MMP7 and MMP9 in NSCLC**. **(A)** The concentrations of CTHRC1 and the serial MMPs were measured by ELISA. The correlation coefficient of CTHRC1 with serial MMPs were shown (n=92). (**B**) Primary tumor tissues were collected from 20 NSCLC patients. The expression of CTHRC1 and MMPs in the tissues was measured by reverse dot hybridization. The correlation coefficient of CTHRC1 with serial MMPs were shown. (**C, D**) Immunohistochemistry demonstrated the expression of CTHRC1 in primary tumor tissues was in associated with the expression of MMP7 and MMP9 (n=230).


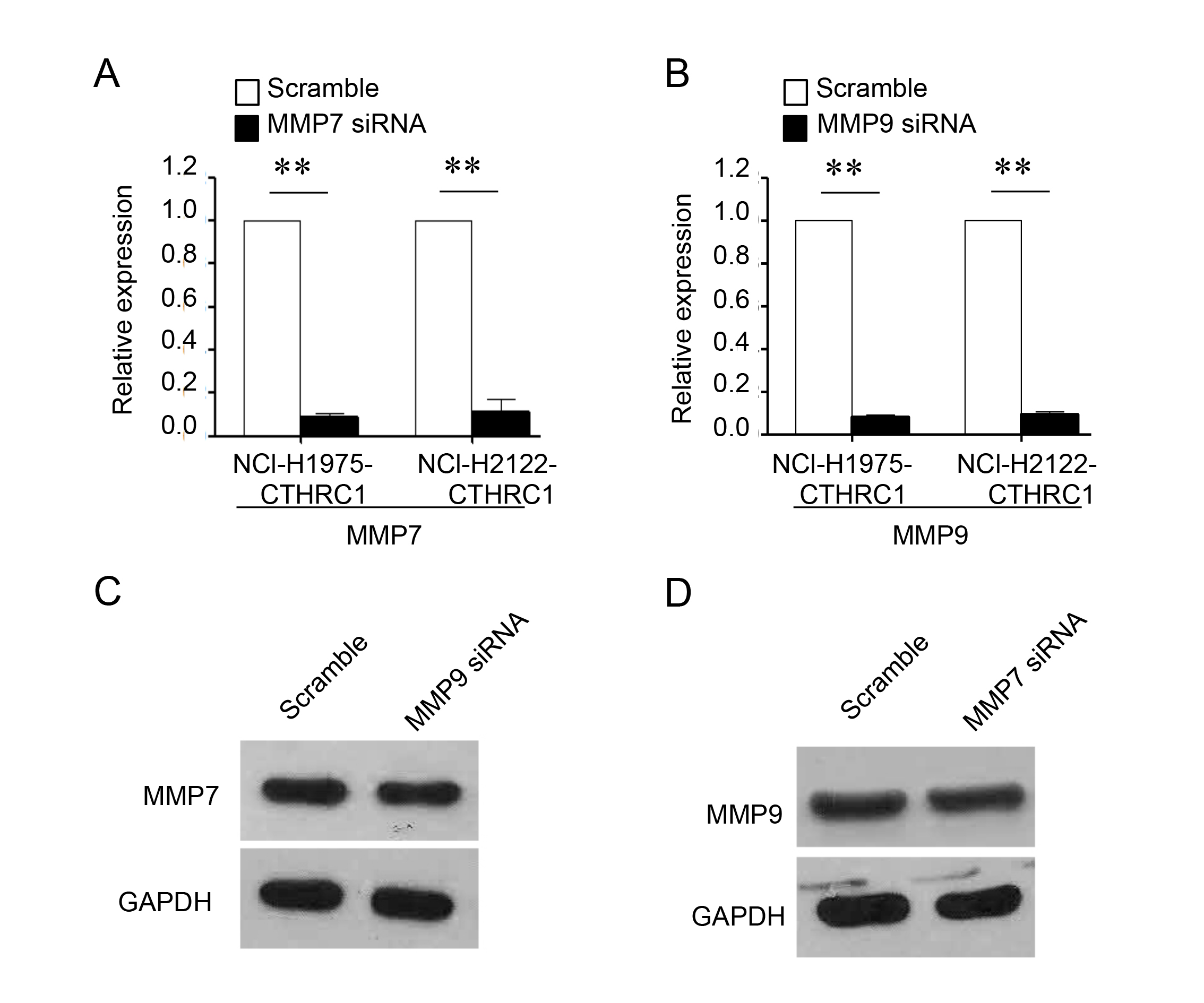


**Figure S4.** **Knockdown of** **MMP7 or MMP9 did not affect MMP9 or MMP7 expression**. **(A, B)** Knockdown efficiencies of MMP7 and MMP9 in NCI-H1975 and NCI-H2122 cells were confirmed by RT-PCR. **(C)** MMP7 was measured by western blotting in MMP9 knockdown or control cells. **(D)** MMP9 was measured by western blotting in MMP7 knockdown or control ***p*<0.01.


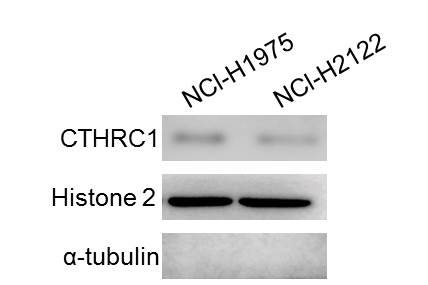


**Figure S5.** **The nuclear localization of CTHRC1 by western blot in NCl-H1975 and NCl-H2122 cells.** Histone 2 was used the inner control of nuclear protein and α-tubulin was used the inner control of cytoplasmic protein. **
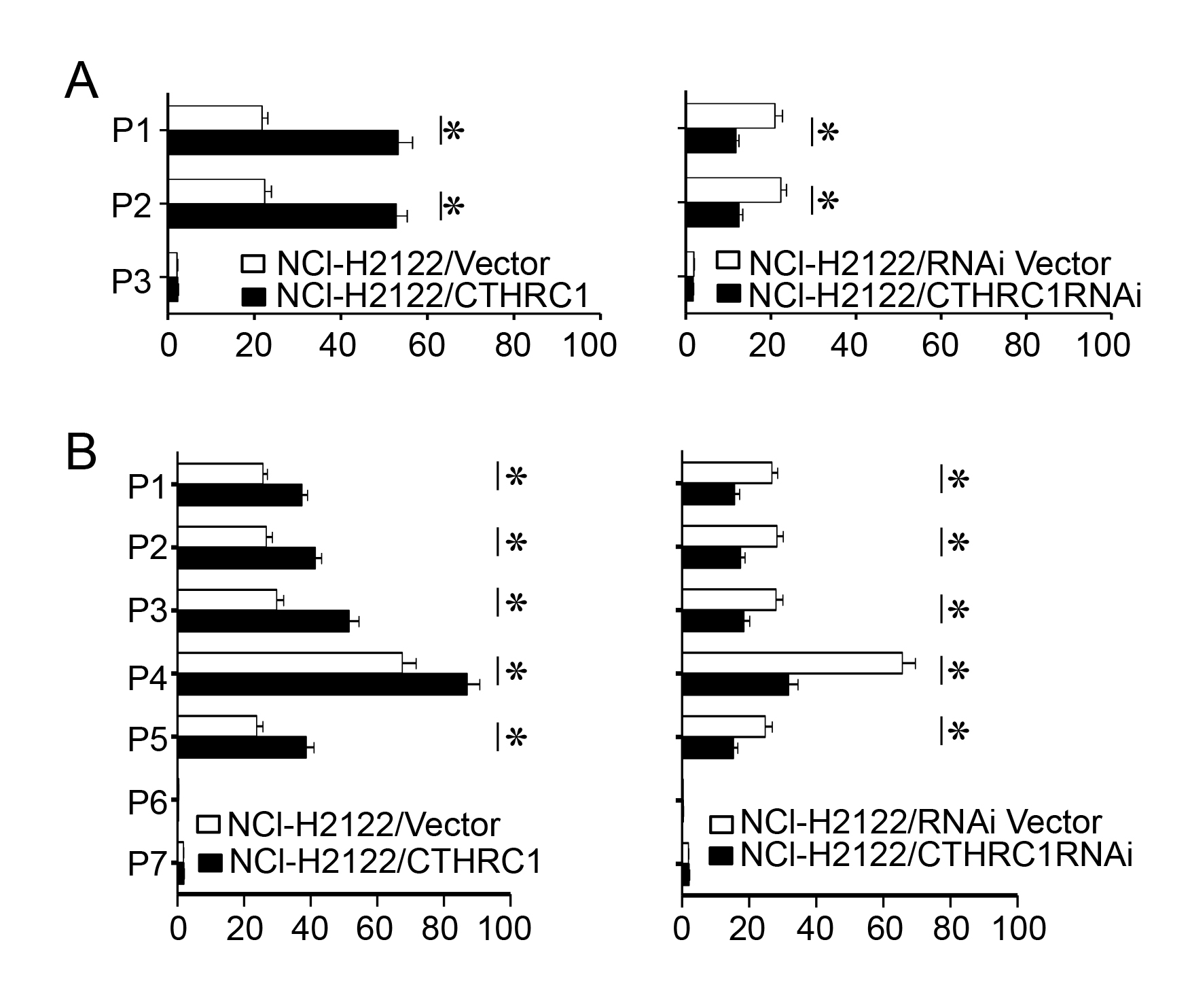
Figure S6.** Transactivating activity of CTHRC1 on serial MMP7 or MMP9 promoter fragments as indicated in NCI-H2122. Overexpression of CTHRC1 enhanced the promoter activities in P1 and P2 in MMP7 promoter region and P1-5 in MMP9 promoter region, while knockdown of CTHRC1 downregulated promoter activities. **p*<0.05.


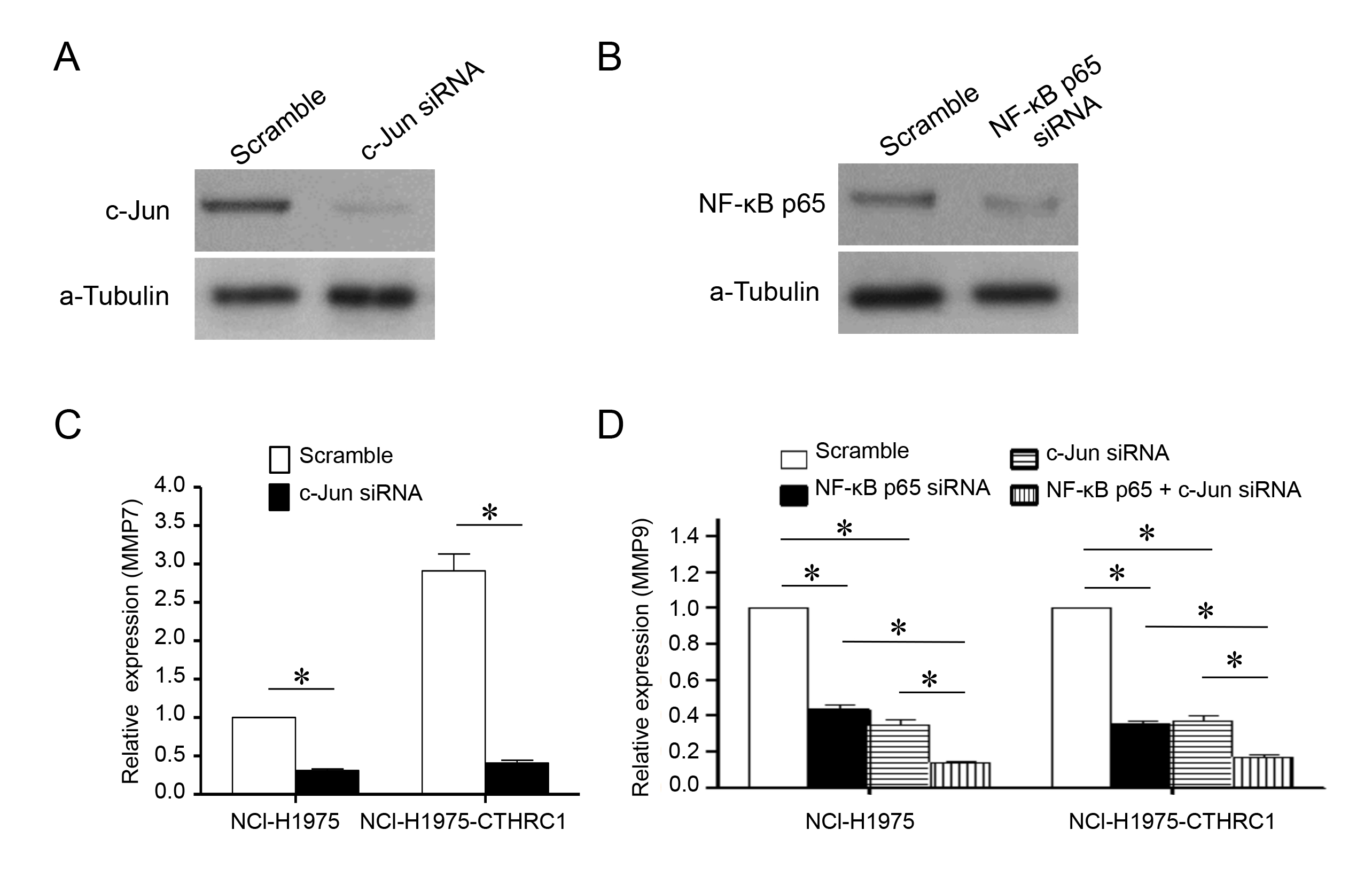


**Figure S7.** Western blottiing demonstrated the knockdown efficiencies of c-Jun (**A**) and NF-κB p65 (**B**). (**C**) MMP7 expression was decreased when c-JUN was knocked down as measured by RT-PCR. (**D**) MMP9 expression was decreased when c-JUN or NF-κB was knocked down as measured by RT-PCR. **p*<0.05.


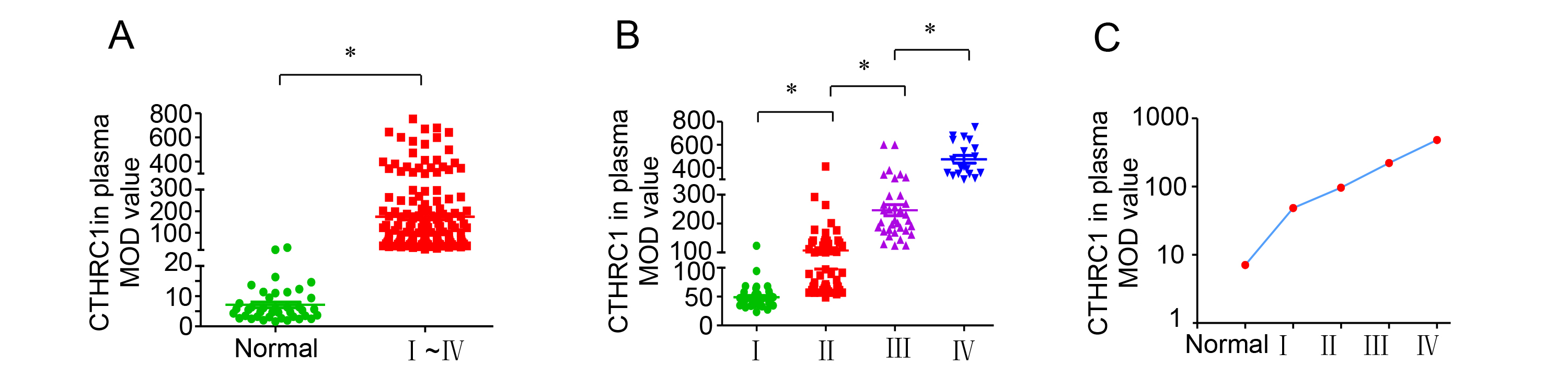


**Figure S8. The elevation of CTHRC1 in NSCLC sera is associated with circulating tumor cells (CTC).** (**A**) CTHRC1 concentrations in the sera of NSCLC patients (n=143) were much higher than those in healthy controls (n=40), based on ELISA analysis. **(B, C)** CTHRC1 concentration in NSCLC was significantly associated with disease stage. **p*<0.05
